# Recent Progress of Structural Design, Fabrication Processes, and Applications of Flexible Acceleration Sensors

**DOI:** 10.3390/s26082499

**Published:** 2026-04-17

**Authors:** Yuting Wang, Zhidi Chen, Peng Chen, Jie Mei, Jiayue Kuang, Chang Li, Zhijun Zhou, Xiaobo Long

**Affiliations:** 1 CTG Wuhan Science and Technology Innovation Park, Wuhan 430014, China; wang_yuting@ctg.com.cn (Y.W.); chen_peng18@ctg.com.cn (P.C.); mei_jie1@ctg.com.cn (J.M.); kuang_jiayue@ctg.com.cn (J.K.); li_chang1@ctg.com.cn (C.L.); 2 China Yangtze Power Co., Ltd., Wuhan 430014, China; zhou_zhijun@ctg.com.cn (Z.Z.);; 3 Hubei Technology Innovation Center for Smart Hydropower, Wuhan 430014, China

**Keywords:** flexible acceleration sensor, intrinsically flexible, sensing mechanism, vibration monitoring

## Abstract

Flexible acceleration sensors demonstrate revolutionary potential in healthcare, structural vibration monitoring, and consumer electronics owing to their unique conformal adhesion capability and mechanical adaptability. However, current academic research presents two distinct paradigms for realizing flexibility: one is the hybridly flexible sensor, which incorporates traditional micro-electro-mechanical System (MEMS) acceleration sensor chips with flexible packaging/substrates; the other is the intrinsically flexible sensor, whose sensing unit and substrate are entirely composed of flexible materials enabled by microstructural design. This review first analyzes the fundamental differences and design challenges between these two flexible architectures. It then systematically elucidates five core sensing mechanisms—capacitive, piezoresistive, triboelectric, piezoelectric, and electromagnetic—comparing their working principles, material systems, structural designs, and performance metrics. Among these, piezoelectric and triboelectric types exhibit distinctive advantages in self-powering capability, whereas resistive and capacitive approaches offer greater ease of integration. Furthermore, the applications of intrinsically flexible acceleration sensors in structural health monitoring, wearable devices, automotive safety, and other fields are discussed, with particular emphasis on their unique strengths in real-time vibration monitoring. Finally, the review summarizes existing challenges, such as the trade-off between sensitivity and flexibility, and provides theoretical insights to guide future innovations in intrinsically flexible acceleration sensor technology.

## 1. Introduction

With the rapid advancement of Internet of Things (IoT), wearable devices, and smart monitoring technologies, acceleration sensors play a vital role in vibration state recognition and fault detection [1]. They are widely applied across fields such as mechanical engineering [2], healthcare [3], vehicle safety [4], consumer electronics [5], seismic prediction [6], and equipment vibration monitoring [7]. By enabling real-time monitoring of frequency and acceleration signals, they facilitate the assessment of operational conditions, thereby enhancing both efficiency and safety [1]. Although traditional micro-electro-mechanical Systems (MEMS) acceleration sensors, based on rigid silicon substrates, offer advantages such as high precision and stability, their inherent mechanical brittleness limits their application in scenarios requiring surface conformability and dynamic deformation. This makes it difficult to meet the demands of emerging applications such as skin-adherent monitoring and vibration measurement on complex curved surfaces [1].

The emergence of flexible electronics technology has opened new development pathways for acceleration sensors. Through material innovation and structural design, devices can achieve stable operational capability under complex deformations such as bending, stretching, and twisting. This advancement addresses the requirements of emerging fields, including wearable health monitoring [8], vibration detection on curved equipment [9], and perception in bio-inspired robots [6]. However, the term “flexible” still lacks a clearly defined standard within the field of acceleration sensors. “Flexibility” encompasses two key aspects: mechanical flexibility and functional flexibility. Mechanical flexibility refers to the device’s ability to withstand deformations such as stretching, compression, and bending without failure; functional flexibility denotes the capacity to maintain stable sensing performance during deformation.

**Figure 1:**
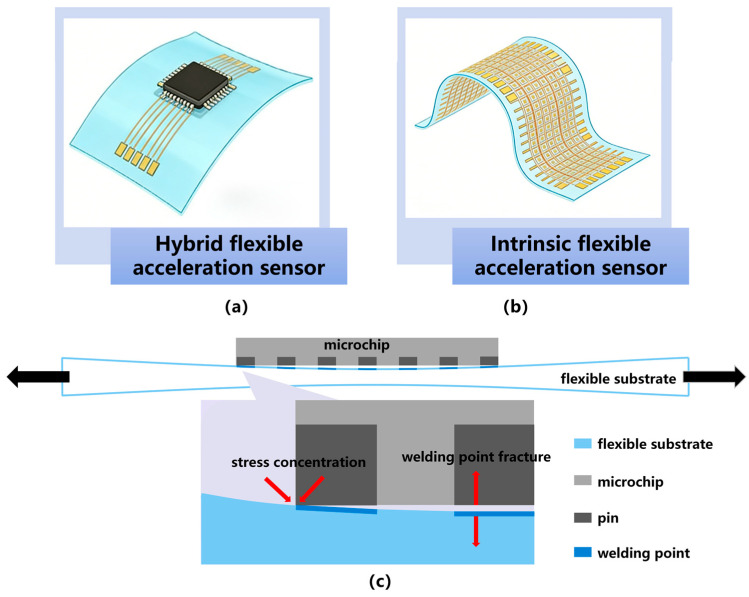
(**a**) Schematic diagram of a hybridly flexible acceleration sensor. (**b**) Schematic diagram of an intrinsically flexible acceleration sensor. (**c**) Schematic illustrating stress concentration and pin-solder joint fracture following repeated bending cycles in hybridly flexible sensors [11].

Current research on the flexibilization of acceleration sensors primarily follows two implementation pathways: hybridly flexible [10] (also referred to as heterogeneously integrated flexible) acceleration sensors and intrinsic-flexible [11] (or all-material flexible) acceleration sensors, as illustrated in Figure 1. Hybridly flexible acceleration sensors utilize conventional high-performance silicon-based MEMS acceleration sensing chips integrated onto flexible printed circuit boards (FPCBs) or polymer substrates, forming a “rigid chip + flexible substrate/encapsulation” architecture [12,13]. Although such sensors can achieve a certain degree of bending, the core sensing unit remains rigid, which restricts their deformation tolerance and conformability to complex curved surfaces. Their bending capability is inherently limited, characterizing them as fundamentally “rigid-flex hybrid” systems. In contrast, intrinsic-flexible acceleration sensors employ flexible components throughout, ranging from sensing materials (e.g., conductive polymers, liquid metals) and functional structures (e.g., microcrack channels, micropillar arrays, porous arrays, seesaw structures) to encapsulation layers, thereby realizing full-material flexibility from the inside out [14]. This design confers excellent bendability, stretchability, and even twistability. In healthcare applications, such sensors enable non-invasive, long-term monitoring of vital signs and movement assessment. In equipment vibration monitoring, intrinsic-flexible acceleration sensors can be directly attached to complex curved surfaces such as pipelines or turbine blades, providing real-time vibration data crucial for predictive maintenance, thus offering a broad application scope [15]. The fundamental distinction between these two types lies in their material systems and failure mechanisms. Hybridly flexible sensors are constrained by stress concentration at the chip-substrate interface, making chips and solder joints prone to fatigue fracture under repeated bending, with low deformation thresholds. Intrinsic-flexible devices, through intrinsic material ductility and optimized mechanical designs (e.g., buckling structures, wrinkle engineering), eliminate rigid-flex boundaries, achieving tensile strain tolerance exceeding 50%.

To achieve high-performance intrinsic flexible acceleration sensing, researchers have explored various theoretical mechanisms, primarily categorized into capacitive [11], piezoelectric [16], piezoresistive [7], triboelectric [17], and electromagnetic types [18]. Each mechanism demonstrates distinct advantages and limitations in sensing principles, material selection, flexible structural implementation, and key performance metrics. For instance, piezoelectric and triboelectric sensors inherently exhibit self-powering potential due to their energy conversion mechanisms, rendering them highly attractive for long-endurance applications; whereas resistive and capacitive types typically offer advantages in terms of circuit integration complexity.

Research and development of intrinsically flexible acceleration sensors confront numerous scientific challenges. Low Young’s modulus of highly flexible materials (0.1–10 MPa) results in insufficient driving force for inertial mass blocks, leading to significantly lower sensitivity compared to rigid MEMS sensors. Multilayer flexible material interfaces are prone to delamination under cyclic deformation. Flexible substrates are susceptible to temperature and humidity variations. Standardized processes have yet to be established for uniform dispersion of nanomaterials [19] (e.g., carbon nanotubes), microstructure lithography/transfer techniques, and flexible packaging technologies. These challenges represent both current research hotspots and critical drivers for technological breakthroughs.

This paper centers on intrinsically flexible acceleration sensors, with the objectives of clarifying the connotation of flexibility and the fundamental distinctions between “hybrid flexibility” and “intrinsic flexibility”. It analyzes the mechanisms enabling flexibility across five categories—capacitive, resistive, piezoelectric, triboelectric, and electromagnetic types—compares material-structure-process co-design strategies, and demonstrates the distinctive advantages of intrinsically flexible sensors in equipment vibration monitoring scenarios through performance comparisons with conventional MEMS devices. Finally, the study examines existing challenges and outlines future research directions, offering a theoretical foundation for the development of next-generation high-performance flexible acceleration sensors.

## 2. Flexibilization Mechanisms of Acceleration Sensors

### 2.1. Hybrid Flexibility of Acceleration Sensors

Hybridly flexible acceleration sensors represent a pivotal transitional form in the evolution of flexible sensing technology. Their defining characteristic lies in the adoption of conventional rigid [10] or semi-rigid MEMS acceleration sensor chips as sensing units [20]. These components are mounted or transferred onto flexible substrates, such as flexible printed circuits (FPC), polyimide (PI), or polydimethylsiloxane (PDMS), to achieve overall flexibility and bendability at the system level. This design strategy ingeniously leverages the advantages of well-established MEMS technology, including high performance, excellent stability, and ease of integration. Meanwhile, innovations in back-end packaging and integration processes enable the sensor device to accommodate a certain degree of deformation—such as bending and twisting—thus making it applicable to non-planar or dynamically changing surfaces [14]. However, the flexibility of such sensors is essentially characterized as “flexible-carrier, rigid-sensing”. The core sensing element itself lacks intrinsic stretchability. Consequently, sensor performance may degrade under repeated or severe mechanical deformation, and its mechanical compatibility with highly compliant surfaces, such as human skin, remains limited.

**Figure 2:**
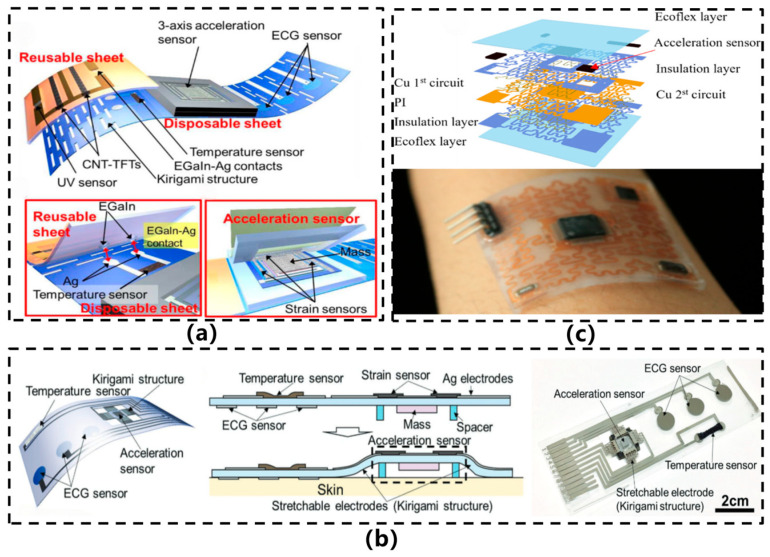
(**a**) A printed multi-functional flexible health monitoring device integrated with motion sensors [10]; (**b**) Schematic diagram of a planar integrated multi-sensing flexible health monitoring patch [20]; (**c**) Exploded view illustrating the operational principle of a flexible wearable acceleration sensor based on an “island-bridge” structure with serpentine interconnects, accompanied by a photograph of the flexible wearable sensor conformally attached to a human arm [21].

Current research in this field primarily focuses on enhancing the overall flexibility and reliability of systems through the optimization of chip thinning techniques, customized substrate designs, and advanced flexible packaging materials and interconnection technologies (such as serpentine wiring), thereby providing an effective technical pathway for high-performance flexible sensing. In 2016, Yamamoto et al. [10] developed a printed multifunctional flexible health monitoring device with an integrated motion sensor, as illustrated in Figure 2a. The device employs a layered, detachable architecture consisting of a disposable sensing sheet based on a polyethylene terephthalate (PET) substrate and a reusable polyimide module. Fabricated via screen-printing on a flexible PET substrate, the sensor structure comprises four beams and three resistive strain sensors utilizing a hybrid ink of silver nanoparticles and carbon nanotubes. Acceleration magnitude is determined by measuring the strain response of the beams. Experimental results indicate that the sensor can detect accelerations within the frequency range of 1–6 Hz, with a threshold of approximately 5–12 m/s^2^ and a maximum sensitivity of 0.064 m/s^2^. However, the sensor exhibits limitations such as relatively low sensitivity, large dimensions, an inability to detect low-intensity movements (e.g., activities of the elderly), and a lack of integration of signal processing and power supply systems. In the following year, Yamamoto et al. [20] further developed a planar-integrated multi-sensing flexible health monitoring patch, as illustrated in Figure 2b. Its acceleration sensing unit operates on the strain-resistive principle, comprising a beam structure and a proof mass fabricated from silver/carbon nanotube composite materials printed on a PET film. To enable out-of-plane vibration space while ensuring wearing comfort, the researchers innovatively incorporated kirigami-inspired electrodes around the sensor. These structures allow the sensing area to gently elevate upon attachment, forming an off-plane vibration gap. By optimizing the beam dimensions, the sensor achieves an acceleration detection threshold below 3 m/s^2^, capable of capturing subtle human activities such as gait patterns in elderly individuals. However, the sensor exhibits significant sensitivity fluctuations under bending conditions, and its resistive sensing mechanism imposes limitations on sensitivity, rendering it unable to detect weak motions like respiration. Moreover, the output signal is susceptible to interference from complex body movements, necessitating further algorithmic processing for accurate interpretation. This study pioneered the monolithic integration of acceleration, temperature, and electrocardiogram sensors on a single flexible substrate via full-printing technology, offering a novel approach to multifunctional health monitoring. Nonetheless, it remains a “hybridly flexible” acceleration sensing strategy reliant on rigid proof masses and substrate flexibility.

He et al. [21] developed a flexible wearable acceleration sensor utilizing an “island-bridge” structure with serpentine interconnects, as illustrated in Figure 2c. The sensor employs a polyimide (PI) substrate as the flexible base, connecting components such as an MPU6050 six-axis MEMS chip and an STM32 microcontroller via serpentine copper traces (18 μm thick). The device is encapsulated in an approximately 1 mm thick Ecoflex elastomer, resulting in overall dimensions of 35 mm × 35 mm. Through structural design, the sensor imparts flexibility to conventional MEMS chips, thereby improving wearing comfort and mechanical adaptability. However, it remains reliant on rigid chips and represents a “hybridly flexible” design, with limited stability and stretchability under extreme deformations. After repeated use, the copper interconnects and chip interface are prone to stress concentration and fracture.

In summary, hybridly flexible acceleration sensors represent a transitional technology involving the heterogeneous integration of conventional rigid silicon-based MEMS sensing chips with flexible substrates such as FPC, PI, and PDMS. Their core structure embodies a “rigid chip + flexible packaging” design, which leverages the mature advantages of MEMS technology—such as high performance, stability, and ease of integration. Enhanced flexibility and bendability are achieved through processes including chip thinning, serpentine interconnects, and flexible encapsulation techniques. However, as the sensing unit itself lacks intrinsic stretchability, repeated or severe deformation may lead to stress concentration at the chip-substrate interface, solder joint fatigue, and fracture. This design offers a limited deformation threshold and constitutes a “flexible-bearing, rigid-sensing” semi-flexible solution, making it suitable for applications with moderate deformation requirements, such as wearable health monitoring.

### 2.2. Intrinsically Flexible Acceleration Sensors

The intrinsically flexible acceleration sensor represents a profound practice of flexible electronic technology in the field of sensing. Its core lies in the complete system, from sensitive materials, functional structures, to packaging substrates, all being composed of flexible or stretchable materials, achieving a true “from inside to outside” flexibility. Unlike traditional MEMS or hybridly flexible approaches, the intrinsically flexible sensor completely abandons the rigid silicon-based chips and instead relies on new materials and new structural designs to directly convert mechanical deformation into electrical signals, thus maintaining stable sensing functions in bending, stretching, twisting, etc., under large deformation states.

To achieve full flexibility sensing, the material system of the intrinsically flexible acceleration sensor mostly selects functional composite materials with good extensibility and electrical properties, mainly including: (1) conductive materials, such as silver nanowires, graphene, carbon nanotubes, conductive polymers and liquid metals (gallium indium tin alloy), used to construct flexible electrodes and conductive pathways [22]; (2) dielectric or piezoresistive materials, such as polydimethylsiloxane (PDMS), Ecoflex elastomers and their composite materials with nano-fillers, used to achieve capacitance changes or strain responses [11]; (3) piezoelectric/frictional materials, such as polyvinylidene fluoride (PVDF), barium titanate (BaTiO_3_) nanowires, zinc oxide (ZnO) nanostructures and their polymer composite systems, used to achieve electromechanical conversion and self-powered operation [23]. In terms of structural design, intrinsically flexible sensors often adopt biomimetic or micro-nanoelectronic engineering strategies to balance sensitivity and flexibility. Common structures include: cantilever-mass block structure [24], sandwich-type capacitance stack [11], micro-cracks or micro-grooves conductive networks [25], porous or wrinkled elastic bodies [26], biomimetic cilia/fissure arrays [6], and paper-cut/folding-inspired deployable configurations [27]. These designs effectively reduce stress concentration and enhance the stretchability and deformation recovery ability of the devices.

The advantages of the intrinsically flexible acceleration sensor are significant: Firstly, it has excellent conformal adhesion ability, which can closely adhere to complex surfaces such as skin and curved devices, enabling in situ, real-time vibration monitoring. Secondly, it has high deformation tolerance, can withstand repeated bending, stretching, and even folding, suitable for dynamic wearables and flexible integration scenarios. Moreover, the piezoelectric and frictional mechanisms give it the potential for self-powered operation, facilitating the construction of wireless, low-power sensing systems. However, the intrinsically flexible design also faces a series of challenges. The low Young’s modulus of flexible materials leads to insufficient inertial driving force, and the sensitivity is usually lower than that of rigid MEMS devices. The multi-layer flexible interfaces are prone to delamination or fatigue under cyclic deformation. Environmental temperature, humidity, and chemical media can easily affect the material stability and signal reliability. Micro-nano processing and integration processes are not yet standardized, and manufacturing consistency, yield rate, and cost control remain bottlenecks for industrialization.

In summary, the intrinsically flexible acceleration sensor achieves a breakthrough in traditional sensor morphology through the collaborative innovation of materials and structures, providing a new solution for achieving biocompatible, wearable, and curved conformal vibration monitoring. However, its performance and reliability still need to be continuously optimized in material engineering, structural mechanics, and integration processes to promote its transition from the laboratory to practical engineering applications. Table 1 summarizes the comprehensive comparisons between hybrid flexible sensors and intrinsic flexible sensors in all aspects.

## 3. Classification of Flexible Acceleration Sensors

### 3.1. Capacitive Acceleration Sensors

Capacitive sensing represents a critical and extensively investigated methodology in flexible acceleration sensors. Its operational principle is derived from the classical parallel-plate capacitor formula, whereby mechanical signals are transduced into electrical signals by detecting changes in the electrode separation distance or effective overlap area resulting from the displacement of a proof mass under acceleration. In flexible configurations, this approach typically employs a “sandwich” or “cantilever-mass” architecture. The primary challenge lies in constructing high-performance variable capacitor elements using compliant materials, such as dielectric elastomers (e.g., PDMS, Ecoflex) and flexible electrodes (e.g., silver nanowires, graphene, conductive textiles). Capacitive sensors are renowned for their inherent advantages, including high sensitivity, low power consumption, excellent temperature stability, and minimal noise, making them particularly suitable for detecting weak low-frequency vibration signals. Nonetheless, their susceptibility to electromagnetic interference and potential signal drift remain key challenges that must be addressed in the design process.

**Figure 3:**
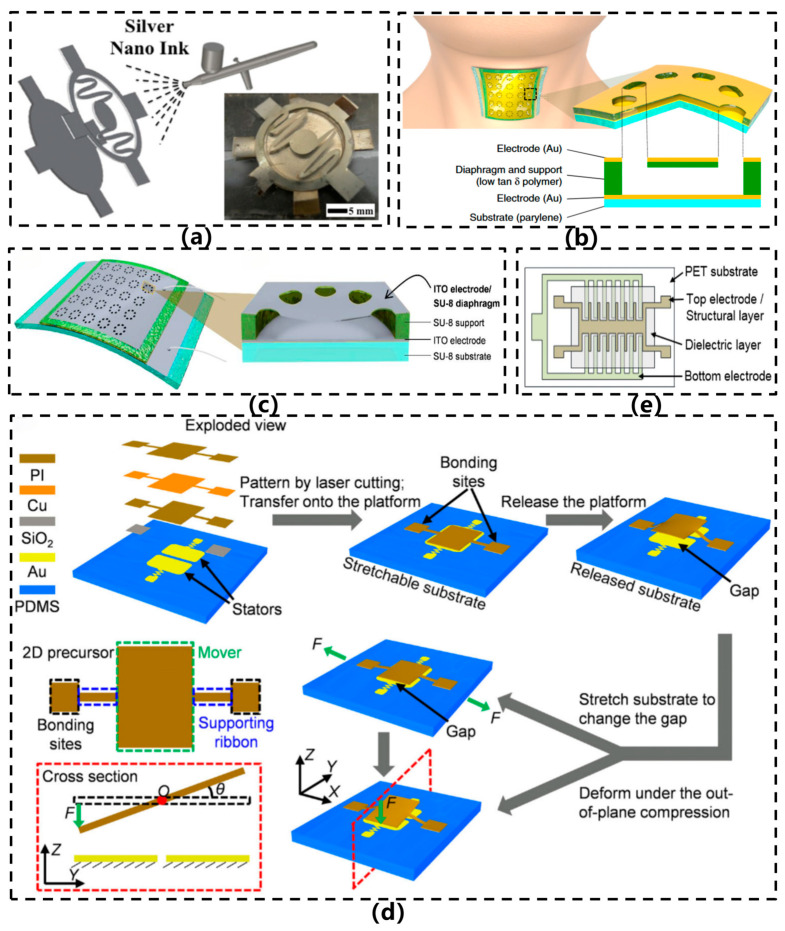
(**a**) Flexible paper-based acceleration sensor fabricated via silver nano-ink printing technology [28]. (**b**) Ultrathin skin-conformal capacitive electronic skin sensor for vibration monitoring [11]. (**c**) An array of transparent and bendable vibration sensors with a corresponding cross-sectional view [29]. (**d**) Schematic diagram of a flexible capacitive sensor based on an adjustable-gap 3D seesaw structure [30]. (**e**) Working principle schematic of a comb-structure acceleration sensor [31].

Current research focuses on innovative microstructural designs (e.g., porous/micro-pyramidal dielectric layers), the introduction of novel nanocomposite materials to enhance dielectric constants, and the development of differential capacitive circuits to improve noise immunity—collectively advancing their application in high-precision human–machine interfaces and health monitoring systems. In 2013, Zhang et al. [28] developed a paper-based flexible capacitive acceleration sensor, as depicted in Figure 3a. This sensor employed silver nanoink as the electrode material and utilized spray-coating and laser-cutting techniques to fabricate a cantilever-based parallel-plate capacitive structure on a cellulose paper substrate. An elliptical bridge structure was incorporated to support the proof mass, thereby improving sensitivity. Within a z-axis acceleration range of 1–10 g, the device achieved a sensitivity of 20 fF/g and exhibited a resonant frequency in the range of 90–171 Hz. By integrating paper substrates with printing technologies, this design realized a flexible, lightweight, and cost-effective acceleration sensing architecture. In 2019, Lee et al. [11] developed an ultrathin, skin-conformable capacitive electronic skin sensor for speech recognition, as illustrated in Figure 3b. This sensor employs a fully cross-linked SU-8 epoxy resin as the flexible diaphragm material, with each diaphragm structure incorporating eight micropore patterns fabricated using micro-nanofabrication techniques such as photolithography, thermal evaporation, spin coating, and transfer printing. With a total thickness of less than 5 μm, the sensor exhibits excellent flexibility and superior skin conformability. By detecting skin acceleration on the neck, the device demonstrates a flat frequency response and high sensitivity across the 80–3400 Hz range. Through the suppression of mechanical resonance and air damping via a low-damping polymer and microporous structure, the study achieved high-fidelity, environmentally robust quantitative speech recognition. Subsequently, Lee et al. [29] developed a transparent and flexible capacitive vibration sensor, as illustrated in Figure 3c. This sensor employs an organic–inorganic hybrid wheel-shaped thin-film structure composed of SU-8 polymer and ITO electrodes, fabricated through processes including photolithography, sputtering, HCl vapor etching, and low-temperature annealing. With an overall thickness of approximately 20 μm, it exhibits high transparency (>80% in the visible light range) and excellent flexibility, allowing it to conform to curved surfaces. The sensor demonstrates a flat frequency response within the 80–3000 Hz range, with a sensitivity of 20 mV/g and a linear measurement range of 0.2–2 g. This research achieves the integrated realization of high transparency and flexible vibration sensing, making it suitable for applications such as skin-adhered voice detection and vibration monitoring in handheld tools.

In 2021, Ye et al. [30] developed an intrinsically flexible capacitive sensor based on a tunable-gap 3D seesaw structure for force and acceleration measurement, as illustrated in Figure 3d. The sensor was fabricated using laser cutting, electron beam evaporation, and pre-stretched PDMS substrate transfer techniques to construct a multilayer PI/Cu/PI cantilever-based differential capacitive structure. Its sensitivity can be continuously tuned over a wide range by mechanically adjusting the initial electrode gap, achieving a maximum tuning ratio of 33-fold. The sensor demonstrates an acceleration sensitivity of up to 0.197 pF/g and a force resolution as high as 5.22 nN. Through structural design, it achieves tunability and compatibility between sensitivity and measurement range, overcoming the conventional trade-off between sensitivity and range in traditional sensors. However, it is only suitable for low-frequency vibration measurements and relies on an external stretching mechanism for adjustment, indicating room for improvement in system integration and dynamic response performance. In 2022, Lee et al. [31] successfully fabricated a flexible comb-type capacitive acceleration sensor utilizing roll-to-roll gravure printing technology combined with a transfer bonding process, as illustrated in Figure 3e. The sensor comprises upper and lower sections: the lower section consists of fixed fingers and a dielectric layer, while the upper section incorporates a movable mass, movable fingers, and a spring structure. Both sections were separately printed on flexible PET substrates and subsequently bonded to form an air gap. With dimensions of 18.5 × 12.0 mm^2^ and an initial capacitance of 7.54 pF, the device exhibited a favorable linear response within the 0–2.0 g range, achieving a sensitivity of 0.00133%/g. The sensitivity here refers to the rate of relative capacitance change per gram, that is $(\Delta C/C_{0})/g$. This study realized a printed air-gap structure without the need for etching processes and demonstrated the potential of roll-to-roll fabrication for high-throughput production of flexible sensors.

In summary, the capacitive intrinsically flexible acceleration sensor is based on the principle of parallel plate capacitance. It converts signals through the change in plate spacing or effective area caused by the mass block under the action of acceleration. Its core advantages lie in high sensitivity, low power consumption, good temperature stability, and low noise characteristics, making it suitable for weak low-frequency vibration monitoring. However, this mechanism is susceptible to electromagnetic interference and suffers from signal drift problems. Recent research has focused on microstructure innovation (such as porous/micro- pyramid dielectric layers), the application of new nanocomposite materials, and the design of differential capacitance circuits to enhance anti-interference ability and measurement accuracy. Representative works include paper-based cantilever beam structures, ultra-thin skin-contact sensors, adjustable gap seesaw structures, and fully printed comb-shaped electrodes, demonstrating the wide application potential of capacitive sensors in health monitoring, speech recognition, and industrial vibration detection.

### 3.2. Resistive Acceleration Sensors

The resistive (or piezoresistive) sensing mechanism has become one of the mainstream technical routes for realizing intrinsically flexible acceleration sensors due to its intuitive principle, simple readout circuit, and ease of system integration. Its core principle relies on the piezoresistive effect, where the resistivity of the sensitive material changes with the applied stress or strain. When acceleration causes deformation of the flexible cantilever beam or support structure, the embedded or constituting piezoresistive material (such as carbon nanotube/polymer composites, graphene foam, metal nanoparticle films, silicone rubber-based composite elastomers, etc.) will undergo corresponding resistance changes. By measuring this resistance value, the acceleration can be calculated. The performance of these sensors is highly dependent on the sensitivity coefficient of the piezoresistive material itself and the structural design of the elastic substrate. Resistive sensors typically have the advantages of fast response, strong output signal, and ease of measurement, but they may face issues such as temperature sensitivity, hysteresis, and power consumption during static measurements.

**Figure 4:**
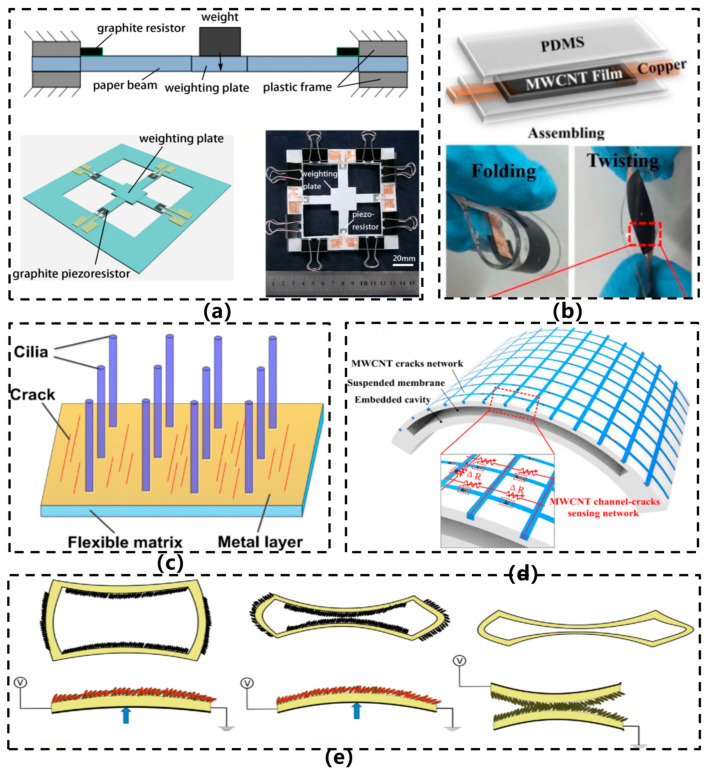
(**a**) Schematic illustration of the structural configuration and device layout for the paper-based sensor [24]. (**b**) Intrinsically flexible resistive vibration sensor based on multi-walled carbon nanotube/polydimethylsiloxane (MWCNT/PDMS) thin film [3]. (**c**) Spider-inspired piezoresistive slit sensilla counterpart model [6]. (**d**) Intrinsically flexible vibration sensor incorporating a suspended sensing membrane with channel-cracked MWCNT/PDMS architecture [25]. (**e**) Resistive vibration sensor utilizing a porous PDMS/graphene composite structure [32].

Current research frontiers focus on significantly enhancing device sensitivity and linearity through strategies such as designing multi-level microcrack structures, anisotropic nanonetworks, or three-dimensional porous conductive foams, to meet the demands of diverse application scenarios ranging from human motion capture to industrial equipment health monitoring. In 2012, Ren et al. [24] proposed an inherently flexible paper-based resistive force sensor, as illustrated in Figure 4a. The sensor utilizes standard A4 paper (88 μm thick) as a flexible substrate, with scissor-cut cantilever beam structures. Graphite is deposited using a pencil to form piezoresistive elements, while copper foil electrodes are integrated with silver paste to reinforce connection points. The device is finalized through baking at 80 °C. It demonstrates a sensitivity of 0.9 mV/mN, a force detection range of 50 mN, and a resolution of 500 μN, exhibiting excellent linear response. This approach achieves an ultra-low cost (approximately $0.01 per device), rapid fabrication (<1 h), and a flexible sensor architecture, eliminating the need for cleanroom environments or sophisticated equipment. Furthermore, by incorporating a cavity structure into the sensor design, the core component exhibits vertical deformation under vibration within the cavity, enabling acceleration measurement and laying the groundwork for the development of cavity-based acceleration sensor configurations.

In 2020, Huang et al. [3] developed a flexible resistive vibration sensor based on a multi-walled carbon nanotube/polydimethylsiloxane (MWCNT/PDMS) thin film, as illustrated in Figure 4b. The sensor was fabricated using a vacuum filtration method to prepare the MWCNT layer, which was then encapsulated with PDMS to form a sandwich-like structure, offering high flexibility and wearability. It exhibits exceptional sensitivity, with a response time of less than 20 ms and a frequency detection range of 25–100 Hz. Compared with a standard acceleration sensor, the measurement error is below 1%. This study realized an intrinsically flexible sensor architecture characterized by high sensitivity and rapid response, suitable for conformal integration on complex surfaces and human motion monitoring, enabling effective tracking of joint movements and laryngeal vibrations. In the same year, inspired by the auditory system of spiders, Liu et al. [6] developed a flexible resistive vibration sensor, as illustrated in Figure 4c. This sensor employs PDMS as the substrate, where cobalt particles are self-assembled under magnetic guidance to form a biomimetic ciliary array. A pre-bent platinum layer deposited via magnetron sputtering introduces crack structures, resulting in a sensitive unit with synergistic cilia-crack coupling. The device achieves a gauge factor of up to 150, a vibration sensitivity of 0.5 mV/g, and an effective frequency response range of 0–100 Hz, enabling stable monitoring of low-frequency signals such as human motion, respiration, and seismic vibrations. In 2021, Chen et al. [25] proposed a flexible vibration sensor featuring a multi-walled carbon nanotube (MWCNT)/PDMS suspended sensing membrane structure based on channel-crack design, as illustrated in Figure 4d. This sensor employed a doctor-blade coating process to infuse MWCNTs into pre-fabricated PDMS microchannels, forming a continuous conductive pathway via pre-stretching-induced through-thickness crack structures, followed by bonding onto a flexible cavity substrate to constitute an out-of-plane oscillating suspended membrane. The device demonstrated exceptional performance, with a broad frequency response range of 0.1–20,000 Hz, an acceleration detection range of 0.24–100 m/s^2^, high sensitivity (with a gauge factor up to 593.3), excellent signal-to-noise ratio (40–60 dB), along with robust mechanical stability and repeatability. By utilizing a “channel-confined crack” architecture, the sensor achieved an optimal balance between high sensitivity and stability. Composed entirely of flexible materials, it is suitable for in situ vibration monitoring on human bodies and curved surfaces.

**Figure 5:**
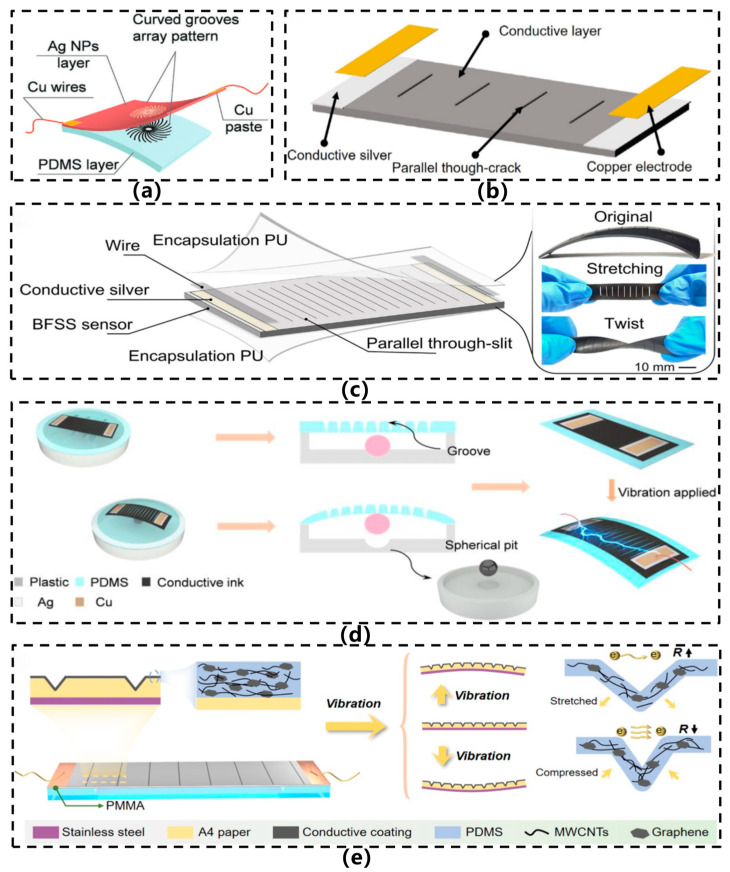
(**a**) Bio-inspired omnidirectional highly sensitive flexible resistive strain sensor [33]. (**b**) Crack-based composite flexible vibration sensor with integrated superhydrophobic properties [7]. (**c**) Schematic diagram and digital image of the bio-inspired flexible strain sensor based on BFSS [34]. (**d**) Three-dimensional model of a flexible vibration sensor based on the piezoresistive effect, depicting its state under vibration excitation [35]. (**e**) Intrinsically flexible vibration sensor employing a paper-based rigid-flex composite structure [9].

In 2022, Korrapati et al. [32] proposed a resistive vibration sensor based on a porous PDMS/graphene composite structure, as illustrated in Figure 4e. The sensor was fabricated using a 3D-printed water-soluble PVA template method to produce porous PDMS foam with a uniform pore architecture. Subsequently, a swelling–dipping–shrinking process was employed to form graphene/PDMS composite flake-like microstructures on the pore walls. This sensor exhibits remarkable strain-rate adaptive behavior, with its gauge factor (GF) increasing significantly as the strain rate rises—achieving up to a 700% enhancement within the strain rate range of 10–1000 mm/min. The device offers a vibration sensing bandwidth of 1–1000 Hz, an acceleration detection range of 0.01–1.01 g, and the capability to discriminate between human motions and mechanical vibrations under varying motion rates. In the same year, inspired by scorpion slit sensilla, Liu et al. [33] developed an omnidirectional, highly sensitive, flexible resistive strain sensor, as illustrated in Figure 5a. Employing a bionic design, this sensor features radially arranged curved microgrooves (Ring Array Around a Central Circle, RACC) surrounding a central circular region on a PDMS substrate, with sputtered silver nanoparticles forming conductive pathways. The fabrication process involves PET template laser-cutting, PDMS transfer imprinting, and metallic sputtering. The sensor exhibits exceptional sensitivity, achieving a gauge factor (GF) exceeding 1400 within the 0–0.46% strain range and surpassing 18,000 in the 0.46–0.65% strain range. It is capable of detecting vibration amplitudes as low as 5 μm, with an operational bandwidth of 70 Hz and cyclic stability exceeding 7000 cycles.

In 2024, Zhang et al. [7] developed a crack-based composite flexible vibration sensor with integrated superhydrophobic properties, as illustrated in Figure 5b. This sensor employs femtosecond laser direct writing technology to fabricate parallel penetrating crack structures on a PDMS film, followed by spraying a conductive ink containing multi-walled carbon nanotubes (MWCNTs), carbon black (CB), and PDMS to form the conductive layer. The sensor exhibits outstanding performance, achieving a frequency response of up to 2000 Hz, an acceleration detection range of 100 m/s^2^, a superhydrophobic contact angle of 159.61°, and a linearity of 0.9812 between voltage and acceleration. It also maintains stable operation underwater. This study integrates femtosecond laser precision machining with a superhydrophobic conductive coating, enabling reliable vibration monitoring in humid and underwater environments, while offering a simple fabrication process and low cost. In the same year, inspired by the slit sensilla of scorpions, Wang et al. [34] proposed a bioinspired strain sensor (Bio-inspired Flexible Strain Sensor, BFSS) with selective responsiveness, leveraging hysteresis effects and a parallel through-slit structure, as illustrated in Figure 5c. The sensor utilized a flexible conductive polymer composite composed of monolayer graphene and styrene-isoprene-styrene (SIS) block copolymer, with parallel through-slit arrays fabricated on its surface via laser cutting technology. The BFSS exhibited high sensitivity (gauge factor up to 657.36), a broad frequency response range (up to 103 Hz), and exceptional frequency discrimination capability (resolution of 0.2 Hz), enabling distinction of vibrational signals with varying frequencies, amplitudes, and waveforms. This work synergistically exploited the intrinsic hysteresis of materials and biomimetic structural design to achieve both high sensitivity and selective frequency response.

In 2025, Mou et al. [35] developed a flexible vibration sensor based on the piezoresistive effect, as illustrated in Figure 5d. Its sensing layer comprises a composite conductive material of Ag/PDMS/MWCNTs/CB, with parallel microgroove arrays fabricated on the surface via femtosecond laser ablation and template transfer techniques to establish conductive pathways. The sensor structure incorporates a stainless-steel ball as an inertial mass, housed within a PLA casing featuring spherical confinement pits, which effectively enhances sensitivity and restricts lateral displacement. The sensor exhibits excellent linearity (correlation coefficient of 0.92) across a vibration frequency range of 50–400 Hz and an acceleration range of 1–5 g, demonstrating stable performance in detecting equipment vibrations and mobile phone ringtones, along with omnidirectional and long-range response capabilities. In the same year, Zhang et al. [9] were inspired by spider slit sense organs and developed a flexible vibration sensor based on a paper-based rigid-flexible composite structure, as shown in Figure 5e. The sensor was constructed with an A4 printed paper, stainless steel sheet, and PET double-sided tape as the laminated substrate, and a parallel V-shaped micro-groove array was fabricated on its surface by femtosecond laser processing. Subsequently, an MWCNTs/graphene/PDMS composite conductive layer was sprayed to form a sensitive path. The sensor demonstrated stable responses within a vibration frequency range up to 800 Hz and an acceleration range of 5 g, and was capable of effectively monitoring the vibration states of equipment such as motors, electric drills, and shield machine cutter heads.

In summary, the resistive intrinsically flexible acceleration sensor utilizes the piezoresistive effect, where the material resistance changes with stress, converting mechanical deformation into electrical signals. Its prominent advantages include a simple structure, fast response, strong output signal, and ease of system integration. However, it also has issues such as temperature sensitivity, hysteresis, and static power consumption. Current research has significantly improved the sensitivity and linearity of the device by designing micro-crack structures, anisotropic conductive networks, three-dimensional porous foams, etc. From the early paper-based graphite sensors to the recently inspired by biology, such as cilia-crack cooperative structures, channel crack suspended membranes, and porous graphene composites, resistive sensors have demonstrated excellent applicability and stability in human motion monitoring, industrial equipment vibration detection, and extreme environment perception.

### 3.3. Triboelectric Acceleration Sensors

Triboelectric acceleration sensors operate based on the principles of contact electrification and electrostatic induction [36], directly converting mechanical vibration into electrical signals with inherent energy harvesting capability. These devices typically consist of two flexible materials with markedly different electron affinities (e.g., PDMS and PET), where microstructural designs (such as pyramid or micropillar arrays) enhance contact area and charge transfer efficiency. Characterized by their self-powering nature, simple architecture, and high impact resistance, they are particularly suitable for vibration monitoring in extreme environments. Nevertheless, their output signals are susceptible to humidity, material wear, and contact consistency issues. Additionally, they exhibit higher sensitivity to vertical acceleration with notable limitations in cross-axis sensitivity [17].

**Figure 6:**
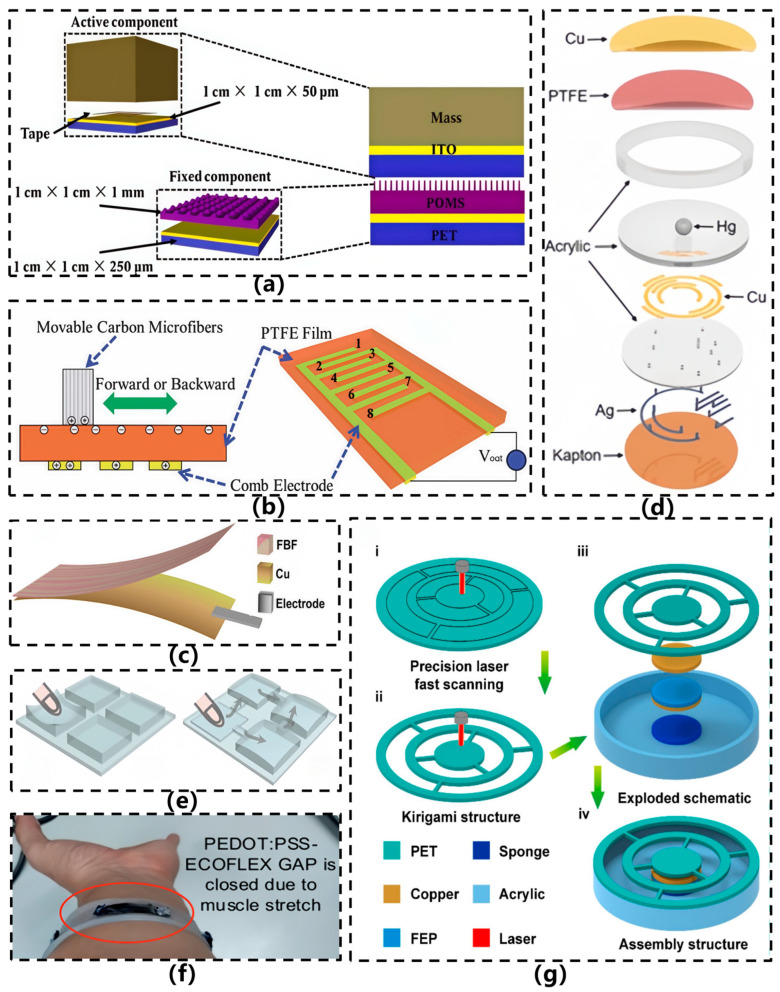
(**a**) Schematic illustration of a flexible acceleration sensor based on PDMS pyramidal microstructures [17]. (**b**) Schematic of a flexible hybrid triboelectric-electret nanogenerator utilizing interdigital electrodes and PTFE electret film [37]. (**c**) Schematic diagram of a flexible triboelectric acceleration sensor employing swim bladder film [38]. (**d**) Schematic of a flexible three-dimensional acceleration sensor based on a liquid metal-based triboelectric nanogenerator [39]. (**e**) Closed flexible interconnected triboelectric nanogenerator array [40]. (**f**) Schematic representation of a flexible forearm triboelectric sensor [22]. (**g**) Intrinsically flexible triboelectric nanogenerator based on kirigami-inspired architecture [27].

In recent years, research efforts domestically and internationally have primarily focused on optimizing microstructure morphology, developing novel flexible triboelectric layer materials, and incorporating multi-modal coupling mechanisms to enhance environmental adaptability and signal stability [17]. In 2017, Xiang et al. [17] designed a flexible acceleration sensor based on PDMS pyramid microstructures using a contact area variation approach instead of conventional spring structures, as illustrated in Figure 6a. The core configuration comprises a PDMS layer featuring pyramid microstructures and a PET/ITO electrode layer. The flexible triboelectric layer, fabricated via wet etching of silicon molds and subsequent PDMS casting, exhibits pyramid spacing of 10 μm and 20 μm. This sensor eliminates the need for traditional spring-beam structures; instead, it relies on mass-induced variations in contact area between the PDMS and PET layers under acceleration, generating triboelectric charges and outputting electrical signals. Capable of withstanding high-g impacts and featuring a compact form factor, this sensor provides a novel approach to the design of elastic space configurations. In 2018, Zhu et al. [37] proposed a flexible hybrid triboelectric-electret nanogenerator based on interdigital electrodes and electret PTFE film for self-powered tracking of position, motion direction, and acceleration, as illustrated in Figure 6b. The sensor employs a corona-charged PTFE film as the negative charge layer, copper interdigital electrodes as the sensing layer, and carbon microfiber bundles as the external friction material, operating in a planar sliding mode. Experimental results demonstrate that after corona charging, the short-circuit current (~0.6 μA) and open-circuit voltage (~11.2 V) increased approximately 3-fold and 6-fold, respectively, compared to the uncharged state. The output exhibited a numerical correlation with acceleration within the range of 0.1–5 m/s^2^, indicating excellent motion sensing capability. By integrating triboelectric and electret enhancement effects, this intrinsically flexible, highly sensitive device enables multi-parameter sensing without an external power supply.

In 2020, Ma et al. [38] proposed a flexible triboelectric acceleration sensor based on fish bladder film (FBF-TENG), as illustrated in Figure 6c. The sensor utilized a natural fish bladder membrane as the triboelectric layer, on which copper nanoparticles were directly deposited to form electrodes, resulting in an ultrathin (0.08 mm), ultralight (0.0968 g), and bendable single-electrode structure capable of flexing up to 360°. By employing collagen-derived biomaterials, the sensor achieved excellent biocompatibility, biodegradability, and a high dielectric constant. It demonstrated linear response to acceleration (sensitivity ≈ 0.45 μA·s^2^/m) and humidity, while also enabling non-contact position detection within a range of 0–27 mm. The sensor has been successfully applied in fields such as robotic distance perception and wireless smart switching systems. In 2021, Zhang et al. [39] proposed a flexible three-dimensional acceleration sensor based on a liquid-metal triboelectric nanogenerator (LM-TENG), as illustrated in Figure 6d. The sensor employs a mercury droplet as a movable triboelectric unit, forming a contact-separation structure with a PTFE film surface-modified with nanowires. Signal acquisition and directional recognition are achieved via a laser-cut acrylic substrate and encoded copper electrodes. The cylindrical device (28.75 mm in diameter, 7.22 mm in height) weighs only 3.63 g and exhibits a broad detection range of 0–100 m/s^2^ in the horizontal direction and 0–50 m/s^2^ in the vertical direction, with a sensitivity of 800 mV/g. Moreover, the voltage output shows negligible attenuation after 100,000 cycles. By leveraging the flow characteristics of liquid metal and an electrode encoding strategy, the sensor enables high-precision 3D vector acceleration sensing while maintaining self-powering capability. However, the use of mercury entails toxicity and environmental concerns, and the relatively complex structure may limit its applicability and safety in certain flexible scenarios. In the same year, Lv et al. [40] proposed a sealed flexible interconnected triboelectric nanogenerator (TENG) array for stable mechanical energy harvesting and sensing in humid or even underwater environments, as illustrated in Figure 6e. The sensor employs silicone rubber as the flexible encapsulation substrate, with films featuring lotus-inspired biomimetic microstructures and sandpaper-textured silicone rubber serving as the positive and negative triboelectric layers, respectively. Unit structures were fabricated using laser engraving and casting techniques, while interconnected air channels were designed to optimize internal pressure distribution. Through full encapsulation and interconnected airway design, the sensor significantly enhances environmental adaptability, output stability, and sensitivity (0.37 V/kPa), demonstrating successful applications in gait monitoring and self-powered waterproof keyboards.

**Figure 7:**
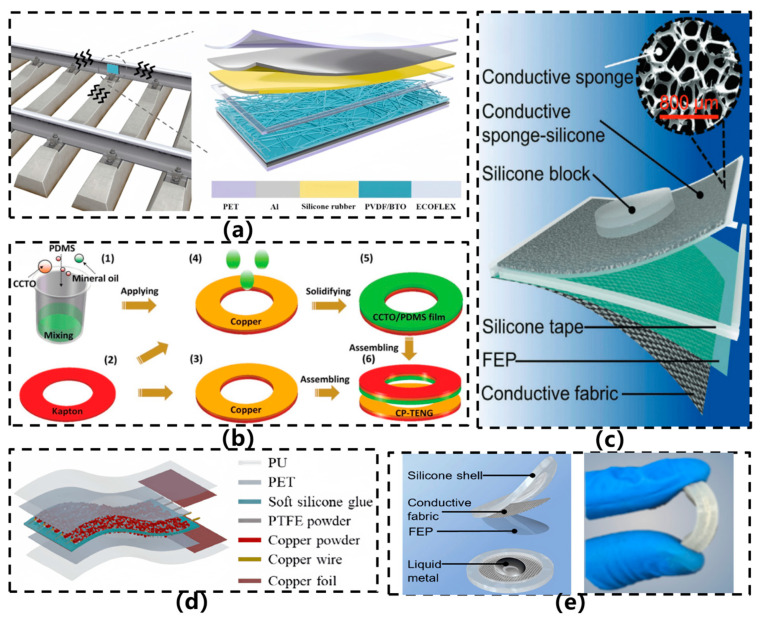
(**a**) Schematic diagram of a self-powered flexible vibration sensor fabricated from electrospun nanofibers [23]. (**b**) CCTO/PDMS-based triboelectric nanogenerator with annular configuration [41]. (**c**) Schematic of a conductive sponge silicone sensor [26]. (**d**) Schematic illustration of an ultrathin grid-structured flexible triboelectric acceleration sensor [42]. (**e**) Schematic of a highly sensitive flexible triboelectric vibration sensor utilizing liquid metal [43].

In 2022, Vera et al. [22] developed a flexible triboelectric forearm sensor for diagnosing and monitoring Parkinson’s disease (PD), as illustrated in Figure 6f. The sensor, entirely constructed from flexible materials using Ecoflex™ and PEDOT:PSS as triboelectric layers, operates in a contact-separation mode. It generates electrical signals by detecting subtle displacements of the skin surface (with gap variations of approximately 0.5 cm) caused by forearm muscle and tendon movements during finger and hand motion. Measuring 3 cm × 1 cm, the device offers excellent conformability and comfort, with an output voltage range spanning from tens of millivolts to several volts. It is capable of distinguishing various amplitudes and frequencies of hand tremors and bradykinesia—characteristic symptoms of PD—while supporting motor assessments based on the MDS-UPDRS scale. By combining triboelectric sensing with indirect motion capture, the design eliminates the need for direct sensor placement on the hand, thereby improving long-term wearability. In the same year, Qi et al. [27] proposed a flexible triboelectric nanogenerator based on a kirigami-inspired structure (KI-TENG) for ultrawide-band vibrational energy harvesting and self-powered acceleration sensing, as illustrated in Figure 6g. This sensor utilizes a laser-cut PET film as the substrate, where an origami-inspired structural design replaces conventional springs to realize elastic systems with one or two degrees of freedom. Copper and FEP serve as triboelectric layers, with plasma etching applied to enhance surface charge density. Under vertical vibration conditions, the device achieves energy harvesting across a frequency range of 2–49 Hz, generating an output voltage of several tens of volts. It is capable of monitoring acceleration variations from 1 to 9 m/s^2^, demonstrating sensitivities of 3.6 V/(m/s^2^) and 17.5 V/(m/s^2^) at 15 Hz, respectively. Meng et al. [23] developed a self-powered flexible vibration sensor (SNVS) based on electrospun nanofibers for safety monitoring of rail fastener tightness, as illustrated in Figure 7a. Featuring an intrinsically flexible structure, the sensor employs BTO doping to modulate the β-phase content of PVDF (up to 91.9%), substantially enhancing triboelectric output. This design enables a linear response to rail vibration acceleration (0–1.5 g) and real-time discrimination of fastener tightness levels.

With the expanding application scope of flexible acceleration sensors, devices tailored for specific scenarios have gradually emerged. In 2023, Sun et al. [41] proposed a CCTO/PDMS-based triboelectric nanogenerator (CP-TENG) featuring a ring-shaped structure, designed to achieve self-powered acceleration sensing with intrinsic flexibility and to address the installation challenges associated with conventional sensors, as illustrated in Figure 7b. This sensor employs CCTO/PDMS composite film and copper foil as the triboelectric pair, where the dielectric properties are enhanced by doping CCTO particles into the PDMS matrix. The device is fabricated by integrating a photoresist annular substrate with patterned copper foil onto a gasket structure, enabling direct installation on automotive bolts. Experimental results demonstrate that the sensor exhibits significant output voltage and current responses within an acceleration range of 9–60 m/s^2^, and is capable of identifying bolt loosening conditions through signal characteristics, showcasing high sensitivity, excellent temperature stability, and reliability over nearly ten thousand operational cycles. In 2024, Zou et al. [26] developed a flexible self-powered triboelectric vibration sensor for mechanical condition monitoring, which demonstrated outstanding performance in curved-surface detection scenarios, as shown in Figure 7c. The sensor comprises a conductive sponge–silicone composite layer and a fluorinated ethylene propylene (FEP) film, both fabricated entirely from flexible materials. By infusing silicone into the conductive sponge, an integrated electrode–triboelectric structure was formed, while a flexible conductive fabric served as the counter electrode, resulting in holistic intrinsic flexibility. The sensor is capable of detecting accelerations ranging from 5 to 50 m/s^2^ and vibration frequencies from 10 to 100 Hz, maintaining stable output even after 168,000 cycles, thereby exhibiting exceptional durability. It enables high-fidelity vibration monitoring on curved surfaces, with linear fitting degrees reaching 0.954 and 0.989 under planar and curved conditions, respectively, highlighting its strong adaptability. In 2025, Wu et al. [42] developed an ultrathin grid-structured flexible triboelectric acceleration sensor (UGS) for foot integration and vibration source detection and localization in quadruped robots, as illustrated in Figure 7d. The sensor, constructed from polyurethane (PU), polyethylene terephthalate (PET), copper powder, and polytetrafluoroethylene (PTFE) powder, adopts a grid-cavity architecture fabricated via a three-step manufacturing process. With a thickness of merely 0.5 mm, it can be directly attached to the curved surfaces of robotic legs. It exhibits a broad frequency response (8 Hz–6 kHz), a sensitivity of 0.49584 mV/(m/s^2^), and maintains stable output after 35,000 cycles of operation. In the same year, Zou et al. [43] developed a highly sensitive, flexible triboelectric vibration sensor based on liquid metal (LM-TENG) for mechanical vibration monitoring, as illustrated in Figure 7e. The sensor employs a eutectic gallium-indium-tin alloy droplet (Ga:In:Sn = 68.5:21.5:10 wt%) as the triboelectric layer, encapsulated between a fluorinated ethylene propylene (FEP) film and a conductive fabric electrode, all embedded within a silicone matrix to form a freestanding layer-structured device. Under optimized parameters (liquid metal mass: 1.5 g, electrode spacing: 2.75 mm), the sensor exhibited outstanding performance: a sensitivity of 0.218 V·m^2^·s^2^, a broad frequency response range (1–5000 Hz), high output linearity (R^2^ = 0.995), and stable operation after 216,000 cycles. Leveraging the rheological properties and interfacial adaptability of the liquid metal, the design enables efficient mechanical-to-electrical energy conversion and conformal sensing on curved surfaces. It has been successfully applied in vibration monitoring of ship blowers and air compressors, demonstrating strong practical engineering applicability.

In summary, the triboelectrically based flexible acceleration sensor converts mechanical vibration directly into electrical signals through the effects of contact electrification and electrostatic induction, and has self-powered characteristics. Such sensors are typically composed of materials with significant differences in electronic affinity, and their charge transfer efficiency is enhanced through microstructure design. The advantages include no need for an external power source, simple structure, strong shock resistance, and suitability for monitoring in extreme environments; however, the output is susceptible to humidity, wear, and contact consistency, and is mostly sensitive to vertical acceleration. The research focuses include optimizing microstructure morphology, developing new friction layer materials, and introducing multi-modal coupling mechanisms to improve environmental adaptability and signal stability. From pyramid microstructures, liquid metal TENG to paper-cut structures, fish bladder films, and other biological materials, triboelectric sensors have shown broad prospects in robot perception, industrial monitoring, and wearable medical applications.

### 3.4. Piezoelectric Acceleration Sensors

Piezoelectric flexible acceleration sensors operate based on the principle that piezoelectric materials generate electric charges under mechanical deformation, with the charge magnitude being proportional to the applied mechanical force [44]. Typically, such sensors incorporate an internal mass that, when the device undergoes acceleration along with the measured object, exerts an inertial force proportional to the acceleration on the piezoelectric element. This force induces charge generation, enabling acceleration detection. These sensors exhibit advantages including high-frequency response, superior signal-to-noise ratio, and self-powering capability, making them particularly suitable for dynamic vibration monitoring. Through structural designs such as laminates, fibers, or porous configurations, piezoelectric output can be enhanced while maintaining flexibility. However, limitations remain, such as relatively low piezoelectric coefficients, susceptibility to fatigue and aging, and insensitivity to static acceleration. Current research focuses on developing flexible composite materials with high piezoelectric constants, constructing biomimetic hierarchical structures to improve sensitivity, and exploring integrated strategies with flexible electronic circuits [45].

**Figure 8:**
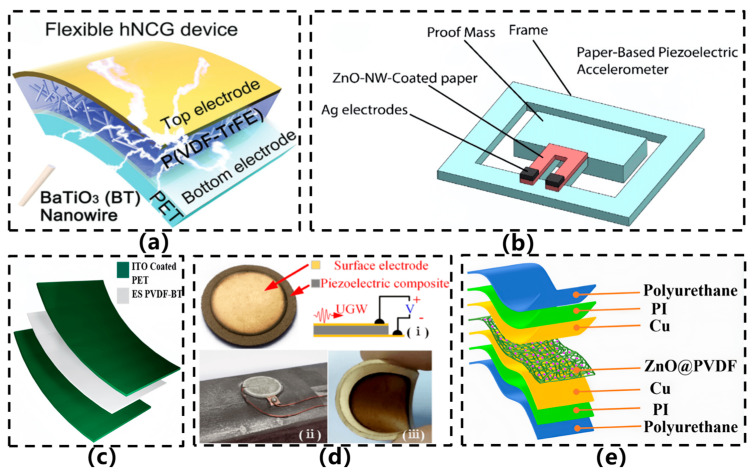
(**a**) Sensor fabricated from an all-piezoelectric nanocomposite comprising barium titanate nanowires (BT NWs) and P(VDF−TrFE) piezoelectric polymer [16]. (**b**) Schematic diagram of a fully paper-based piezoelectric acceleration sensor utilizing cellulose paper and hydrothermally grown zinc oxide nanowires (ZnO NWs) [47]. (**c**) Three−dimensional schematic representation of a flexible piezoelectric nanogenerator (PENG) based on electrospun PVDF-BaTiO_3_ nanofibers [48]. (**d**) Schematic illustration of the flexible piezoelectric sensor employing a novel PZT/silicone resin composite (PZT/SR−FPCS) [49]. (**e**) Schematic depiction of the flexible piezoelectric acceleration sensor based on electrospun ZnO@PVDF composite nanofibers [5].

Piezoelectric materials and devices exhibit excellent manufacturability. Common piezoelectric materials include ceramic-based compositions such as lead zirconate titanate (PZT) and barium titanate (BaTiO_3_), single-crystal materials like quartz (SiO_2_) and lithium niobate (LiNbO_3_), as well as piezoelectric composites [46]. Among conventional rigid acceleration sensors, piezoelectric-based designs are the most widely adopted due to their superior high-frequency response, excellent linearity, and exceptional stability. However, rigid sensors exhibit significant limitations when applied to detect vibrations on curved surfaces in industrial scenarios. Consequently, flexible piezoelectric acceleration sensors have garnered increasing attention from researchers [45]. In 2018, Jeong et al. [16] developed a piezoelectric nanocomposite material based on barium titanate nanowires (BT NWs) and P(VDF-TrFE) piezoelectric polymer, which eliminated the need for non-piezoelectric fillers, for use in high-performance flexible energy harvesters (hNCGs), as depicted in Figure 8a. The sensor was fabricated via hydrothermal synthesis of BT NWs, followed by APTES surface treatment and blending with a P(VDF-TrFE) solution. The device was produced through spin-coating, annealing, and dual-sided polarization processes. It demonstrated outstanding performance, achieving output voltages and currents of up to 14 V and 4 μA, respectively, under bending mode, and effectively responded to human motion such as hand gripping. In the same year, Wang et al. [47] developed a paper-based piezoelectric acceleration sensor utilizing cellulose paper and hydrothermally grown zinc oxide nanowires (ZnO NWs), as illustrated in Figure 8b. This sensor adopted a cantilever-beam configuration, employing a paper-based proof mass and U-shaped ZnO NWs-coated paper as sensing elements, fabricated via laser cutting and screen-printing techniques. It achieved a maximum sensitivity of 16.3 mV/g and a natural frequency of 84.75 Hz, albeit with a relatively narrow operational bandwidth (approximately 17 Hz). The study demonstrated the feasibility of a flexible sensor entirely constructed from paper-based materials, characterized by low cost, biodegradability, and facile fabrication. However, limitations such as relatively low sensitivity, restricted frequency response range, and inconsistent output reproducibility across devices hinder its application in scenarios requiring high-precision measurements or broadband monitoring.

In 2022, a research team led by Athira et al. [48] developed a flexible piezoelectric nanogenerator (PENG) based on electrospun PVDF-BaTiO_3_ nanofibers for self-powered vibration sensing, as illustrated in Figure 8c. The sensor features a sandwich-structured design incorporating ITO-PET flexible electrodes through a thermal lamination process. Under an external excitation of 3 N at 4 Hz, the device achieved an open-circuit voltage of 50 V, a short-circuit current density of 0.312 mA/m^2^, and a power density of 4.07 mW/m^2^, outperforming pure PVDF-based devices by more than tenfold. This study innovatively employs electrospun piezoelectric composites for real-time monitoring of mechanical vibration anomalies in applications such as CPU fans and hard disk drives, demonstrating robust self-powered sensing capabilities. In 2023, Jiang et al. [49] developed a novel flexible piezoelectric composite sensor based on PZT/silicone rubber (PZT/SR-FPCS) for impact monitoring on curved surface structures, as illustrated in Figure 8d. The sensor was fabricated by blending PZT-5A powder with silicone rubber at a 50% volume ratio, followed by processes including vacuum stirring, mold pressing, magnetron sputtering of gold electrodes, and high-temperature polarization. It exhibits excellent flexibility and conformability, with an elastic modulus of only 34.2 MPa, a yield strain of 7.22%, a fatigue strain limit of 9200 µg, and a strain sensitivity ranging from 8.59 to 9.81 mV/µε. The sensor also demonstrates stable Lamb wave detection capability even at 75 °C. The study innovatively proposed an impact localization method based on dominant energy narrowband extraction, the effectiveness of which was verified on an aircraft nose cone curved surface. In 2025, Wu et al. [5] developed a flexible piezoelectric acceleration sensor based on electrospun ZnO@PVDF composite nanofibers, as illustrated in Figure 8e. The sensor adopts a sandwich structure (Cu-PI electrode/piezoelectric fiber membrane/Cu-PI electrode) encapsulated within a thermally shrinkable polyurethane film. With a ZnO doping content of 3%, the β-phase content in PVDF increased to 80.7%, achieving a piezoelectric coefficient d33 of 68.8 pC/N and a sensitivity of 4.558 V/g—12.2 times higher than that of pure PVDF. The sensor also exhibited a favorable frequency response and durability (12,000 cycles) within the 20–50 Hz range. Furthermore, the researchers constructed a self-powered wireless acceleration monitoring system based on this sensor, which was successfully deployed in scenarios such as vehicle collisions and bicycle wheel rotation monitoring.

In summary, piezoelectric intrinsically flexible acceleration sensors achieve acceleration sensing by utilizing the characteristic of piezoelectric materials that generate charges during mechanical deformation. They have advantages such as good high-frequency response, high signal-to-noise ratio, and self-powered operation, and are particularly suitable for dynamic vibration monitoring. However, their piezoelectric constants are usually low, they are insensitive to static acceleration, and are prone to fatigue aging. Current research focuses on developing flexible composite materials with high piezoelectric coefficients (such as PZT/silicone resin, ZnO@PVDF nanofibers), constructing biomimetic multi-level structures to enhance sensitivity, and exploring integration with flexible circuits. From barium titanate nanowire composite materials, all-paper-based ZnO sensors to high-temperature-resistant composite piezoelectric films, piezoelectric sensors have significant application value in structural health monitoring, vehicle collision detection, and wearable energy collection, etc.

### 3.5. Electromagnetic Acceleration Sensors

The operational principle of electromagnetic acceleration sensors is grounded in Faraday’s law of electromagnetic induction, where a change in magnetic flux—induced by relative motion—generates an induced electromotive force in a coil. Implementing such sensors in inherently flexible systems presents considerable challenges, as they typically require the integration of rigid magnets or coil structures. Recent developments have introduced partially flexible designs, utilizing materials such as liquid metal conductors, printed flexible coils, or magnetic elastomer composites, to improve bendability and conformal adhesion of the overall structure. These sensors offer high sensitivity, excellent linearity, and robust reliability while operating without an external power supply. However, they are often constrained by large dimensions, high power consumption, and difficulties in achieving full flexibility [8].

**Figure 9:**
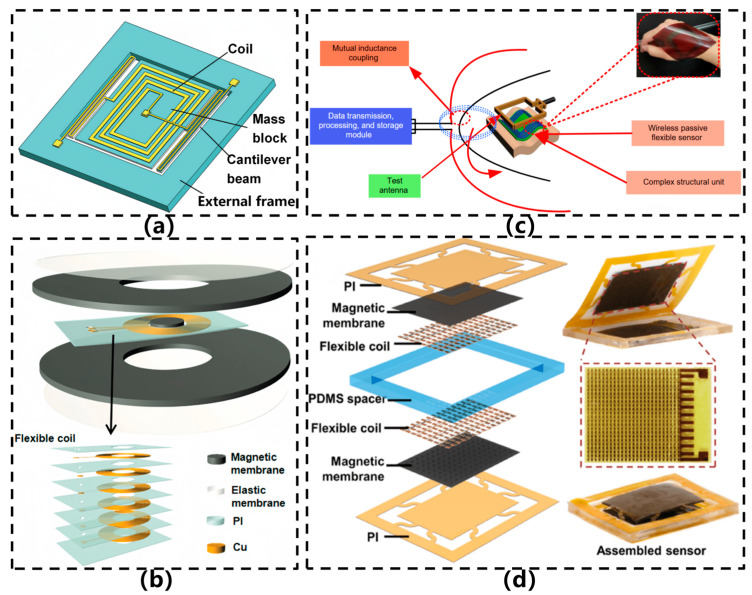
(**a**) Classic structure of an electromagnetic sensor [18]. (**b**) Schematic diagram of a flexible vibration sensor based on electromagnetic induction principles [8]. (**c**) Schematic illustration of a wireless, passive flexible acceleration sensor operating on the LC resonance principle [50]. (**d**) Layer-by-layer structural and assembly schematic of a flexible vibration sensor utilizing the magnetic levitation principle [18].

Current research primarily focuses on miniaturized magnetic circuit design, the development of flexible magnetic composite materials, and heterogeneous integration strategies to maintain performance while enhancing application potential in wearable devices. Figure 9a illustrates a typical structure of electromagnetic sensors [18]. In 2020, Zhao et al. [8] developed a fully flexible vibration sensor based on the principle of electromagnetic induction, as illustrated in Figure 9b. This sensor employs a multilayer flexible coil structure integrated with an origami-inspired annular magnetic membrane, which encapsulates a suspended flexible neodymium iron boron magnet. Through the origami design, directional confinement and enhancement of the magnetic field were achieved (with a 291% improvement), overcoming the limitations associated with conventional rigid magnets in electromagnetic sensors. The sensor exhibits a broad frequency response ranging from 1 Hz to 10 kHz, achieving a sensitivity of 0.59 mV/μm at 1.7 kHz, and supports passive wireless signal transmission. With excellent conformability to curved surfaces and mechanical durability, the sensor demonstrates potential for applications such as human motion monitoring, speech recognition, and equipment condition assessment.

In 2022, Li et al. [50] developed a wireless passive flexible acceleration sensor based on the LC resonance principle, which overcame the problem of easy detachment of traditional wired sensors during vibration, as shown in Figure 9c. This sensor utilized MEMS technology to fabricate planar spiral inductors (300 nm thick) on a polyimide (PI) flexible substrate. Non-contact measurement was achieved through electromagnetic coupling. The change in acceleration was converted into a voltage signal output by altering the resonant frequency through the variation in the distance from the antenna. Within the range of 20–100 m/s^2^, the sensor had a sensitivity of 0.27 mV/(m/s^2^), a repeatability error of less than 0.037%, and demonstrated excellent bending adaptability. In the same year, Zhang et al. [18] developed a flexible vibration sensor based on the principle of magnetic levitation, with both the sensing unit and the substrate made of flexible materials, as shown in Figure 9d. The sensor consists of two layers of NdFeB/PDMS composite magnetic films with surface micro-pyramid arrays. One layer of the film is levitated by magnetic force, and mechanical constraints and flexible connections are achieved through polyimide (PI) films and serpentine interconnect structures. The micro-pyramid structure significantly enhances the local magnetic field, increasing the average magnetic flux density by more than 35%. The sensor integrates a flexible coil array, fabricated using electroplating and etching processes, and can be attached to curved surfaces for operation. Experiments show that the sensor has a frequency response range of 1 Hz to 20 kHz, with a sensitivity of 0.82 mV/μm at 120 Hz, and has been successfully applied to voice acquisition, motion detection, and machine condition monitoring.

The electromagnetic all-flexible acceleration sensor is based on Faraday’s law of electromagnetic induction. It generates an induced electromotive force in the coil through the change of magnetic flux due to relative motion. Implementing it in an all-flexible system is quite challenging, as traditional structures often rely on rigid magnets or coils. In recent years, research has partially achieved the flexibility and conformal adhesion capability of the structure by using materials such as liquid metal wires, flexible printed coils, and magnetic elastic bodies. These sensors have the advantages of high sensitivity, good linearity, and no need for an external power supply. However, they also face challenges such as large size, high power consumption, and difficulty in complete softening. Representative progress includes origami magnetic membrane constrained structures, LC resonant wireless sensors, and magnetic levitation flexible vibration units, which demonstrate the potential of electromagnetic sensors in human motion monitoring, speech recognition, and equipment status assessment.

### 3.6. Summary of Sensing Mechanisms

In summary, this section systematically reviews the five principal sensing mechanisms employed in flexible acceleration sensors: capacitive, resistive, triboelectric, piezoelectric, and electromagnetic. Each mechanism exhibits distinct working principles, material requirements, structural designs, and performance characteristics, making them suitable for different application scenarios. Capacitive sensors offer high sensitivity and low noise, while resistive sensors are characterized by simple readout and fast response. Triboelectric and piezoelectric types possess self-powering capability, rendering them suitable for energy-autonomous systems. Electromagnetic sensors, despite facing challenges in achieving complete flexibility, provide high linearity and reliability. To facilitate a clear comparison of these sensing mechanisms, Table 2 summarizes their working principles, primary advantages, and key limitations, serving as a practical reference for selecting appropriate sensor types based on specific application requirements such as sensitivity, frequency response, environmental adaptability, and power consumption. Table 3 presents a comparison table of the performance of each type of sensor for various categories.

## 4. Applications of Flexible Acceleration Sensors

**Figure 10:**
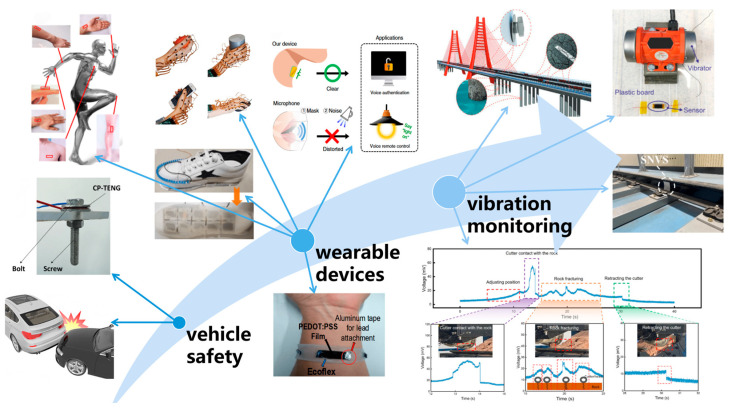
Overview of application domains for intrinsically flexible acceleration sensors.

With continuous breakthroughs in the material and structural design of intrinsic flexible acceleration sensors, their application fields are gradually expanding from traditional rigid scenarios to new scenarios with higher requirements for flexibility and deformation capabilities. These sensors, with their ultra-thin, lightweight, bendable, and stretchable characteristics, can closely adhere to various irregular surfaces, demonstrating significant application potential in vibration monitoring, wearable devices, and automotive safety. In the monitoring of industrial equipment vibration states, they can be directly attached to the curved surfaces of mechanical equipment to achieve long-term, online monitoring of vibrations and impacts, thereby enabling fault prediction. Their excellent environmental adaptability and durability ensure reliable operation in harsh industrial environments. In the field of wearable health monitoring, they can be comfortably attached to the skin or integrated into clothing to continuously capture real-time physiological activity signals of the human body, such as heart rate, pulse, joint movement posture, gait analysis, and even pathological features like Parkinson’s tremors. They can also be applied in the field of speech recognition, providing unprecedented convenience and data accuracy for personal health management, disease diagnosis, and rehabilitation training. In vehicle safety systems, such sensors can be integrated into seats, seat belts, or key parts of the vehicle body in a lightweight and low-power form, not only for collision detection and triggering airbags in passive safety systems, but also for real-time monitoring of vehicle posture and vibration states. Figure 10 summarizes the three application areas of the intrinsic flexible acceleration sensor.

### 4.1. Equipment Vibration Monitoring

Intrinsically flexible acceleration sensors present unique advantages in the field of equipment vibration monitoring. Compared with conventional rigid sensors, which are difficult to install on complex curved surfaces or rotating components and suffer from low measurement accuracy, fully flexible sensors feature ultra-thin, bendable, and conformable designs. These devices can adhere closely to critical parts of machinery like “electronic bandages”, enabling high-precision vibration measurement. They are capable of identifying early-stage fault signatures caused by factors such as mechanical wear, thereby enhancing operational safety and stability. Such sensors have become indispensable across various industrial applications, including bridge monitoring, engine diagnostics, seismic detection, and lathe operation.

**Figure 11:**
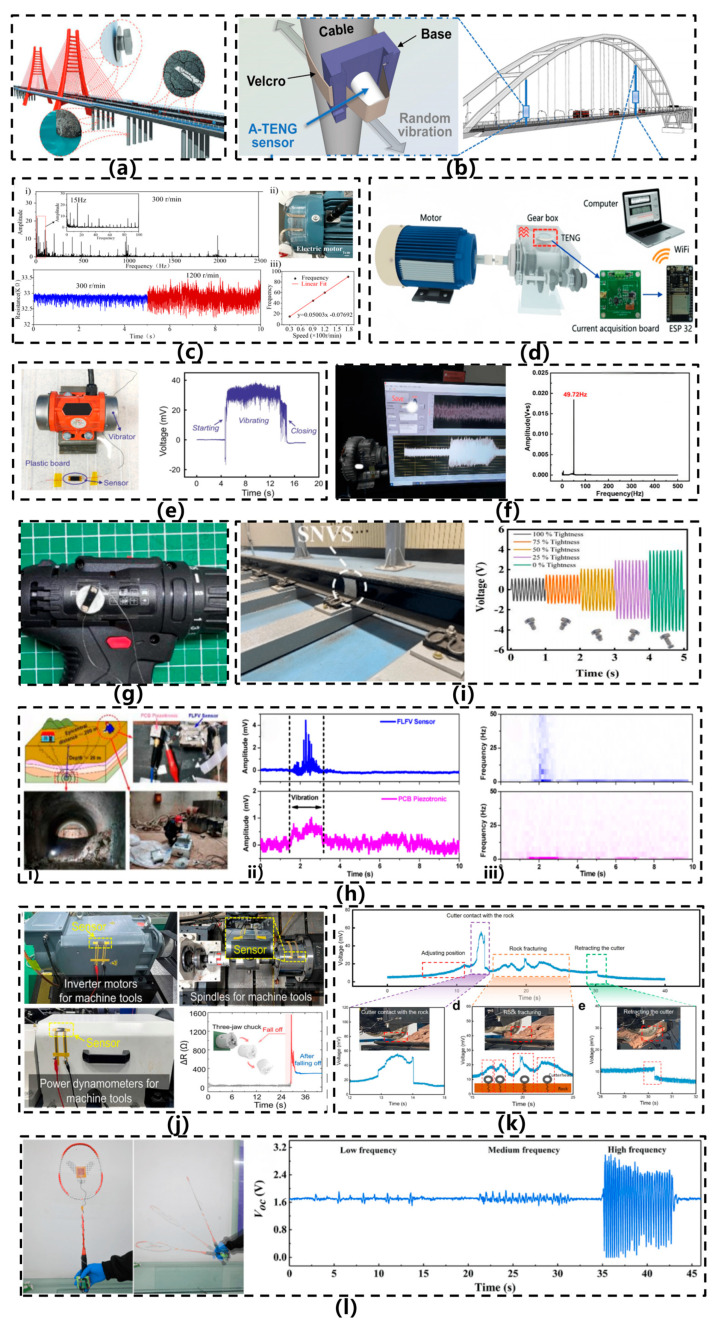
(**a**) A flexible resistive sensor applied for crack monitoring on bridge structures [33]. (**b**) A flexible acceleration sensor deployed for vibration monitoring of bridges [51]. (**c**) A resistive vibration sensor is attached to the curved surface of an electric motor to track vibrations under varying rotational speeds [25]. (**d**) Sensors performing real-time and precise monitoring of mechanical equipment operation status, integrated with embedded systems and wireless transmission technologies [52]. (**e**) Sensors monitor the operational condition of an engine [9]. (**f**) Flexible acceleration sensor utilized for vibration monitoring of blowers in marine applications [43]. (**g**) Sensors are installed on an electric drill to capture vibration signals emitted by the drill bit [35]. (**h**) FLFV sensor based on SCC capable of effectively detecting seismic signals [6]. (**i**) Real-time monitoring of track fastener loosening status via sensors [23]. (**j**) Sensors exhibiting high sensitivity in detecting sudden disturbances occurring in machine tools [34]. (**k**) Sensors capable of distinguishing four operational conditions: cutterhead adjustment, contact between cutterhead and rock, rock fracture, and tool retraction [7]. (**l**) Sensors capturing dynamic acceleration signals generated during rotation or swing motions [5].

Excellent cyclability and high sensitivity are crucial for monitoring vibrations in structural components such as bridges and buildings, as even minor strain variations, including random cracks, corrosion, or loosened bolts, can lead to catastrophic failures. Given that such failures may occur at arbitrary locations, ultrahigh sensitivity and omnidirectional sensing capabilities are essential. In 2022, Liu et al. [33] developed an omnidirectional, highly sensitive, flexible resistive strain sensor inspired by the slit sensilla of scorpions. Mimicking the scorpion’s perception of nearby vibrating objects, the detection process can be divided into three steps: initial vibration detection, identification of the vibration signal characteristics, and localization of the vibration source. A similar procedure can be applied to detect cracks in bridge structures, as illustrated in Figure 11a. Under a strain of 0.65%, the sensor demonstrates outstanding response performance, with response and recovery times of 258 ms and 247 ms, respectively. Figure 11b illustrates a flexible acceleration sensor designed for bridge displacement monitoring, which efficiently converts micro-vibrations of bridge cables into electrical signals [51]. The real-time acceleration time-history curves can be precisely reconstructed from the measured short-circuit current output. In practical applications, these sensors can be deployed on the surfaces of cable structures such as cable-stayed bridges and suspension bridges for extended periods, enabling real-time acquisition of vibration accelerations and fundamental frequencies under environmental excitations, including vehicular traffic and wind loads. The system provides continuous, comprehensive, and reliable data support for cable tension identification, structural health assessment, and fatigue life prediction, thereby substantially enhancing the automation level and data quality of bridge monitoring systems.

Intrinsically flexible acceleration sensors exhibit considerable application potential in engine vibration monitoring. In 2021, Chen et al. [25] mounted a crack-based resistive vibration sensor onto the curved surface of an electric motor to monitor vibrations under varying rotational speeds. As illustrated in Figure 11c, the output resistance waveforms of the vibration sensor at 300 and 1200 rpm demonstrate increasing vibration amplitudes with higher rotational speeds. The frequency spectrum reveals typical vibration characteristics of machinery, including rotational frequencies in the low-frequency range and sideband frequencies. Owing to their flexibility, lightweight nature, and compatibility with curved mechanical structures for in-situ sensing, such vibration sensors hold promising prospects for integration into smart devices. In 2022, Zhao et al. [52] proposed a highly sensitive acceleration sensor capable of real-time and accurate monitoring of mechanical equipment operating conditions, as illustrated in Figure 11d. This sensor is widely applicable for vibration detection in rotating machinery such as gearboxes, air compressors, and heat guns. By analyzing waveform variations during equipment operation, it can accurately identify abnormal conditions (e.g., shaft misalignment, reverse rotation, coupling failures) and, with embedded systems and wireless transmission technology, display processed data in real time on local screens or remote terminals, enabling intelligent diagnostics and early warnings for equipment health [53]. Furthermore, the sensor can be utilized in construction quality inspection—for instance, in detecting hollow tiles—demonstrating its potential in structural health monitoring. Its applications span multiple domains, including gearbox operation monitoring, thermal gun speed variation sensing, and hollow tile detection. In 2025, Zhang et al. [9] attached sensors to the surface of engine vibrators to monitor the operational status of machinery, as illustrated in Figure 11e. Before vibrator activation, the sensor output signal exhibited an initial linear characteristic with constant amplitude. Upon activation, the vibrator generated oscillations, inducing mechanical vibrations in the sensor. The suspended sensitive membrane of the sensor underwent continuous oscillation, resulting in measurable resistance variations. Furthermore, with both the vibrator and sensor mounted on the same plastic substrate, the sensor successfully detected vibration signals emitted by the vibrator during operation. Consequently, through real-time analysis of output signals, this methodology enables the assessment of machinery operational conditions and provides early warnings when trends indicating severe component friction or wear are detected. The flexible acceleration sensor developed by Zou et al. [43] demonstrates significant applicability in vibration monitoring for marine blowers, as depicted in Figure 11f. By adhering directly to the blower’s housing surface, the sensor captures mechanical vibration signals in real time during equipment operation, enabling precise identification of vibration frequencies at varying rotational speeds. For instance, a rated speed of 2980 r/min corresponds to a vibration frequency of approximately 49.72 Hz, showing strong agreement with experimentally measured data. Beyond continuous monitoring of start-stop cycles and operational statuses, the sensor facilitates spectral analysis (via FFT) for the timely detection of anomalous vibration signatures. This capability provides robust data support for fault prediction and preventive maintenance, substantially enhancing the operational safety and reliability of marine equipment. To demonstrate the potential of the designed and fabricated sensor in monitoring the mechanical operating state indicated by vibration, Mou et al. [35] integrated the sensor into a tangible vibration monitoring scenario. As shown in Figure 11g, the sensor was installed on an electric drill, which operated in both clockwise and counterclockwise modes. Regardless of the working mode, the sensor exhibited a distinct response when the drill was activated, transitioning from a stable initial linear signal to one reflecting the vibration signals emitted by the drill bit. Further FFT analysis of the output electrical signal revealed characteristic frequencies of the drill bit operating at approximately 26 Hz, 122 Hz, 29 Hz, and 534 Hz.

In the domain of seismic monitoring, the flexible low-frequency vibration (FLFV) sensor based on sheet with the coexistence of cilia and cracks(SCC) proposed by Liu et al. [6] exhibits a pronounced response to low-frequency vibrations, enabling effective detection of seismic signals and holding significant potential for earthquake early warning systems. As illustrated in Figure 11h, the FLFV sensor was deployed to monitor an artificially induced seismic event resulting from an underground explosion at a subway construction site in Chongqing, China. The explosion center was located approximately 200 m from the test site at a depth of about 20 m. For comparative analysis, a commercial vibration sensor (PCB Piezotronics 356A15, New York, NY, USA) was utilized to simultaneously record vibration signals. The peak signals detected by the FLFV sensor and the commercial sensor measured 4 mV and 1 mV, respectively. The acquired signals enabled the extraction of key parameters such as seismic event duration, number of shock waves, and timing of peak vibrations. Furthermore, short-time Fourier transform analysis was performed on the vibration signals recorded by both the FLFV sensor and the PCB Piezotronic 356A15 system. The FLFV sensor successfully captured nearly all harmonic frequencies of the artificial seismic event within the 0–50 Hz range (indicated in blue), whereas the spectrogram of the PCB Piezotronic 356A15 system exhibited considerable noise across the entire time domain, largely obscuring the vibration signals induced by the artificial earthquake (shown in red). These findings indicate that the FLFV sensor delivers markedly superior performance in low-frequency vibration sensing compared to the commercial sensor. It is well established that non-destructive primary (P) waves travel through the Earth’s crust more rapidly than destructive secondary (S) waves and Rayleigh waves. Consequently, the developed FLFV sensor, characterized by high vibration sensitivity, offers substantial practical value in providing early warnings for destructive seismic waves.

In the realm of track safety, a sensor developed by Meng et al. [23] enables real-time monitoring of the loosening state of rail fasteners, as illustrated in Figure 11i. The sensor can be firmly attached to the rail surface, collecting vibration signals generated during train passages and converting them into electrical outputs. By analyzing variations in vibration characteristics, it accurately assesses the tightness level of fasteners. Capable of stable operation in complex environments, the system triggers early warnings upon detecting abnormal loosening, offering a real-time, efficient, and cost-effective intelligent monitoring solution for railway traffic safety.

In the field of lathe vibration monitoring, in 2024, Wang et al. [34] artificially induced a state of loosened fastening bolts on a three-jaw chuck mounted on the machine. Based on the resistance variation curves output by the sensor, significant discrepancies in resistance change values were observed. This is attributed to the altered vibration behavior of the machine tool during operation, resulting from the loosened chuck bolts. Consequently, the vibration characteristics deviate from those under normal operating conditions, typically manifesting as increased overall machine vibration amplitude and intermittent occurrence of low-frequency, high-amplitude vibration signals. Leveraging its sensitive electrical properties and exceptional flexibility, the sensor enables real-time health monitoring of machine tool operation. To validate the sensor’s capability in detecting sudden mechanical damage, an artificial damage scenario was created. As shown in Figure 11j, all three fastening bolts of the three-jaw chuck were deliberately loosened while the machine tool operated at high speed. After a period, the chuck detached abruptly due to centrifugal force. At the moment of detachment, the sensor’s output resistance exhibited a sharp transient change before gradually stabilizing. These data indicate that the chuck’s detachment induced a substantial instantaneous vibration in the machine, which was detected by the sensor as variations in resistance corresponding to surface vibration signals. Thus, the sensor is demonstrated to be effective in identifying sudden mechanical damage. These findings confirm that the sensor possesses sensitive detection capabilities for abrupt disturbances in machine tools, making it suitable for applications in mechanical equipment health monitoring to assess damage or abnormal operating conditions.

The tunnel boring machine (TBM) is a critical mechanical apparatus in transportation infrastructure construction, as vibration signals at the interface between the cutterhead and rock strata can reflect its operational status and fault conditions. Consequently, monitoring vibration signals during the cutterhead’s operation aids in elucidating the coupling mechanisms between the cutterhead and rock, thereby enabling accurate assessment of its operational performance. As illustrated in Figure 11k, Zhang et al. [7] affixed a custom-developed sensor to the mounting base of a disc cutter to monitor vibration signals generated during rock fragmentation in real time. The vibration sensor converts mechanical vibrations induced by rock-cutting actions into electrical signals, which are then captured by a wireless transmission device and instantaneously relayed to a computer. The experimental results confirm the sensor’s capability to detect mechanical vibration signals of the TBM cutterhead under various operational states. Subsequent analysis of the acquired vibration data allows for the identification of four distinct working conditions: cutterhead adjustment, cutterhead–rock contact, rock fracture, and tool retraction. Thus, the developed vibration sensor demonstrates robust detection performance for low-frequency vibrations produced during rock-breaking processes and enables reliable identification of different operational modes.

Beyond industrial equipment, intrinsically flexible acceleration sensors can be deployed in daily life devices. As illustrated in Figure 11l, the flexible acceleration sensor can be directly attached to surfaces such as bicycle wheels or badminton racket grips. Leveraging its high sensitivity and rapid response characteristics, it captures dynamic acceleration signals generated during rotation or swing motions. The data are transmitted in real time via a wireless sensing system, enabling precise monitoring of motion states, impact intensity, and action frequency [5].

### 4.2. Wearable Health Monitoring

In the domains of speech recognition and human motion health monitoring, intrinsically flexible acceleration sensors can be seamlessly integrated into smart garments, elastic bandages, or directly attached to the skin surface. They enable real-time and continuous capture of various human motion postures and physiological activity signals, and hold significant applications in areas such as speech recognition, voice authentication, medical diagnostics, disease prevention and treatment, and health monitoring.

**Figure 12:**
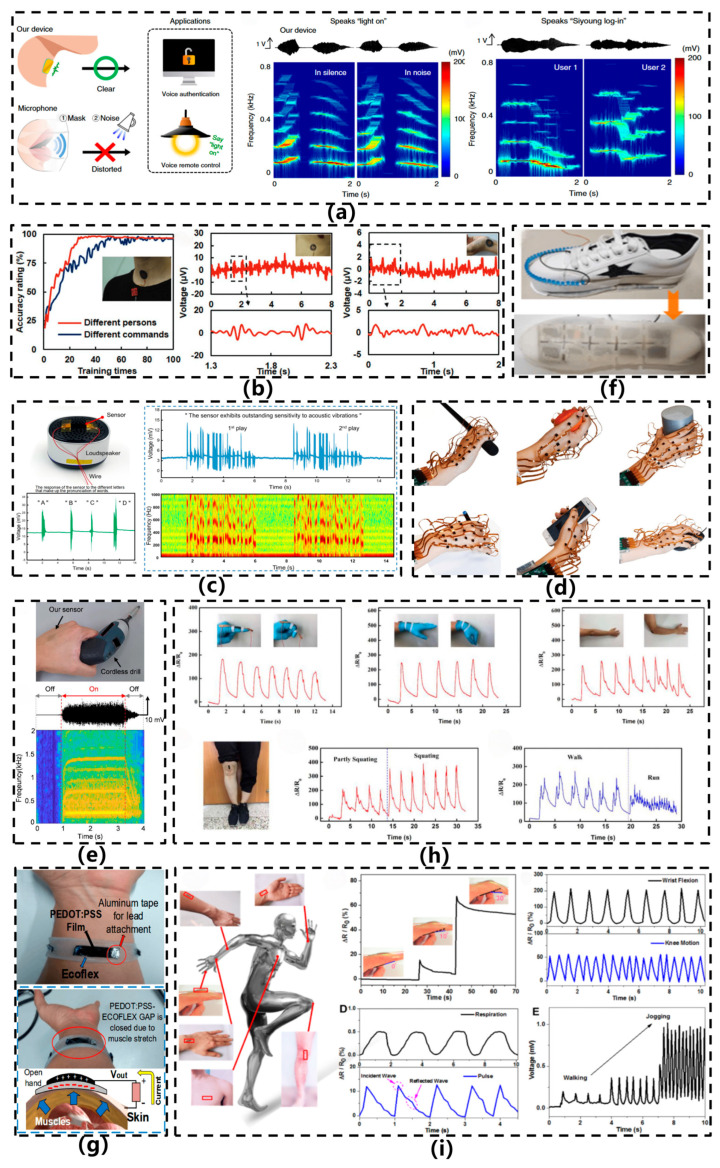
(**a**) Flexible capacitive acceleration sensors find applications in speech recognition and voice-based security systems [11]. (**b**) When attached to the neck skin, such sensors enable laryngeal function monitoring and silent speech interaction interfaces [8]. (**c**) Flexible acceleration sensors demonstrate superior speech recognition capabilities compared to commercial acceleration sensors [7]. (**d**) Hand-mounted flexible acceleration sensors capture distributed vibrational signals during real-time activities, including grasping cups, screen swiping, keyboard typing, and handwriting [54]. (**e**) These sensors monitor hand vibrations to prevent hand-arm vibration syndrome [29]. (**f**) Footwear-integrated flexible acceleration sensors enable real-time gait analysis [40]. (**g**) Flexible acceleration sensors contribute to early Parkinson’s disease diagnosis and personalized rehabilitation management [22]. (**h**) MWCNT/PDMS composite sensors track human motion across subtle to intensive movement ranges [3]. (**i**) Such sensors facilitate respiratory rate/depth monitoring and arterial pulse waveform detection for health assessment [6].

In recent years, voice recognition systems have been widely implemented for controlling computers and peripherals through voice commands. Biometric identification systems present a promising security approach due to their inherently low risks of loss, sharing, or replication. Voice recognition, as an advanced biometric modality, leverages each individual’s unique vocal pattern and offers practically infinite passphrase variations. However, the effectiveness of such systems is often constrained in acoustically noisy environments. In 2019, Lee et al. [11] proposed a flexible acceleration sensor capable of distinguishing the distinctive waveform and spectrum of an individual’s voice. As illustrated in Figure 12a, the system prevents authentication even if another speaker utters the correct passphrase. Furthermore, owing to its sensing mechanism based on skin vibration, it enables clear voice identification even when the user is wearing a mask, thereby significantly reducing the risk of exposing voice credentials to others. Zhao et al. [8] attached flexible electromagnetic vibration sensor onto the surface of the human larynx to monitor subtle mechanical movements generated by vocal cord vibrations during speech in real time, as illustrated in Figure 12b. During phonation, laryngeal skin displacement occurs in response to vocal fold oscillations, and the sensor transduces these mechanical vibrations into electrical signals, enabling highly sensitive speech acquisition. This sensor not only accurately records vibration patterns corresponding to various voice commands (e.g., “thank you”, “sorry”) but also maintains stable performance under complex environmental conditions. It is applicable in scenarios such as speech recognition, laryngeal function monitoring, and silent speech interfaces, offering a comfortable, unobtrusive, and self-powered sensing solution for voice-assisted technologies, medical rehabilitation, and human-computer interaction systems.

Dynamic and continuous mechanical vibrations are ubiquitous in daily life. However, detecting such subtle dynamic vibration impulses poses significant challenges for flexible sensors. In 2024, Zhang et al. [7] positioned the sensor horizontally on a loudspeaker to monitor surface vibrations in real time, while a computer was programmed to command the loudspeaker to emit the phrase “The sensor demonstrates exceptional sensitivity to acoustic vibrations” twice. The output response of the vibration sensor is depicted in Figure 12c. The figure illustrates the sensor’s recorded signals as the loudspeaker sequentially produced the sounds corresponding to the letters A, B, C, and D, demonstrating the sensor’s outstanding capability in detecting and identifying transient acoustic vibrations. Furthermore, the vibration sensor also exhibited the ability to detect and differentiate words with varying numbers of syllables. The speaker repeated the monosyllabic word “one”, the disyllabic word “sensor”, and the polysyllabic word “sensitivity” three times each. The corresponding output responses of the vibration sensor and a commercial acceleration sensor are shown in Figure 5d and Figure 5e, respectively. The results indicate that the electrical signal waveforms generated by the vibration sensor correspond precisely to the syllable count in each word, highlighting its superior speech recognition performance. In contrast, the commercial acceleration sensor, fabricated from rigid materials, fails to maintain conformal contact with the measured object and is unable to recognize polysyllabic words. Therefore, flexible vibration sensors, characterized by their excellent flexibility and superior speech recognition capabilities, hold great promise for widespread application in human–machine interaction scenarios involving voice control.

In the domain of human health monitoring and motion sensing, Shao et al. [54] introduced a flexible acceleration sensor designed for hand applications. This device captures distributed vibrational signals across the entire hand in real time during activities such as grasping a cup, swiping a touchscreen, typing on a keyboard, and writing, as illustrated in Figure 12d. By leveraging distinctions in signal characteristics, it accurately discriminates among different hand gestures, thereby providing realistic tactile feedback for grasping and touch interactions in virtual environments. Furthermore, the system quantitatively records detailed hand vibration data—including frequency and amplitude—generated when users interact with objects of varying materials or surface textures. In the development of robotics and prosthetics, the tactile data collected from natural human tool-grasping and slip detection can serve as a reference for designing tactile interfaces in robotic hands. This enables robots or prosthetic devices to emulate the remote vibration perception capabilities of the human hand through distributed sensing, ultimately achieving more dexterous manipulation. In 2021, a sensor developed by Lee et al. [29] demonstrated the potential of wearable and flexible vibration sensors for vibration-related healthcare monitoring. The sensor, mounted on the dorsum of the hand via attachment to a cordless drill as depicted in Figure 12e, features high transparency and a skin-compatible design when in contact with human skin. Through subsequent vibration measurements of the components transmitted from the drill bit to the hand, the output electrical signals—both in waveform and frequency spectrum—revealed that during drill operation, vibration signals centered around 220 Hz, along with their harmonics (approximately 440, 660, 880, 1100, and 1320 Hz), were transmitted to the hand. According to the UK Health and Safety Executive (HSE), workers should implement technical and organizational measures to reduce exposure once hand-arm vibration exceeds 2.5 m/s^2^, to prevent adverse health effects. Consequently, the developed sensor, by monitoring vibrations transmitted to the hand or body, can help prevent conditions such as hand-arm vibration syndrome or whole-body vibration phenomena. In the same year, Lv et al. [40] proposed a flexible acceleration sensor integrated into shoe insoles for real-time gait monitoring, as illustrated in Figure 12f. This system displays distinct voltage distributions corresponding to the gait patterns of different wearers when the insoles are worn. Due to substantial variations in gender, height, weight, and walking habits among individuals, the resulting voltage distributions also differ. When wearers walk or run in low-light conditions, the smart footwear can accurately identify the wearer by detecting approaching vehicles, thereby offering technological support for applications in sports, healthcare, and related fields. In 2022, He et al. [22] attached flexible acceleration sensor onto the surface of the forearm skin of Parkinson’s disease patients, as illustrated in Figure 12g. By capturing subtle deformations of muscles and tendons during actions such as finger flexion-extension and wrist rotation in real time, the method enabled quantitative monitoring of core symptoms, including tremor and bradykinesia. The hand opening-closing and finger tapping movements were converted into electrical signal outputs, thereby accurately reflecting variations in motion amplitude, frequency, and rhythm. This approach supports long-term, non-intrusive at-home symptom monitoring and provides physicians with objective, continuous assessment data for the disease condition, contributing to early diagnosis and personalized rehabilitation management of Parkinson’s disease. In 2020, Huang et al. [3] affixed MWCNT/PDMS sensors onto volunteers as wearable devices to monitor human motion ranging from subtle to strenuous activities. When joint movement occurs, deformation is transmitted to the thin-film sensor, and the piezoresistive effect of the conductive network is utilized to capture recordable resistance change signals. The MWCNT/PDMS sensors were fixed at the knee joint to monitor various motion states, such as walking, running, and squatting, as illustrated in Figure 12h. The ΔR/R0 ratio of the MWCNT/PDMS strain sensor increases upon knee flexion, with its amplitude reflecting the degree of bending. Furthermore, distinct waveform variations and frequency shifts between walking and running can be clearly discerned. Owing to the sensor’s high flexibility and sensitivity, minor peaks appear in the resistance response, attributed to slight vibrations caused by articular deformation during motion. It was observed that the resistance changes by several hundredfold with each joint movement. Under varying magnitudes of dynamic mechanical strain, the signals consistently exhibited a high signal-to-noise ratio, which is crucial for human motion monitoring applications.

In 2020, Liu et al. [6] employed a chest-mounted FLFV sensor to record real-time respiration, as illustrated in Figure 12i. Through frequency analysis, respiratory rate and depth were accurately identified, alongside monitoring of the magnitude of Respiratory Chest Retractions (RCR). The detected respiratory signals not only offer valuable insights into cardiopulmonary function but also serve as an early-warning system for conditions such as Sudden Infant Death Syndrome (SIDS) and adult sleep apnea. Additionally, an FLFV sensor affixed to the wrist of an adult test subject was used to monitor arterial pulse waves. The calculated average pulse frequency was 65 beats per minute, consistent with the typical heart rate of a healthy adult—a finding of significant relevance for cardiovascular disease diagnosis. When mounted on a robotic arm, the FLFV sensor tracked vibrations induced by rolling motions in forward, backward, and lateral directions. Forward rolls resulted in minimal vibration owing to dual-arm protection, whereas backward rolls produced the strongest vibrations due to the absence of such protection. The motion data captured by the FLFV sensor are instrumental for robotic motion and posture control, and the technology also shows potential for extension to applications such as fall detection in the elderly.

### 4.3. Vehicle Safety Monitoring

Flexible acceleration sensors offer a novel technical solution for vehicle safety monitoring by leveraging their thin, lightweight, and bendable properties. These sensors can be conformally installed on curved surfaces of critical structural components, tire interiors, seats, or steering wheels to monitor vehicular vibrations in real time. Compared to conventional rigid sensors, flexible sensors not only adapt to complex surface installations and exhibit high resistance to mechanical fatigue, but also enable distributed perception of vehicle operating conditions. Integrated with the Internet of vehicle systems, they facilitate early warnings and provide data support for proactive safety measures and predictive maintenance.

**Figure 13:**
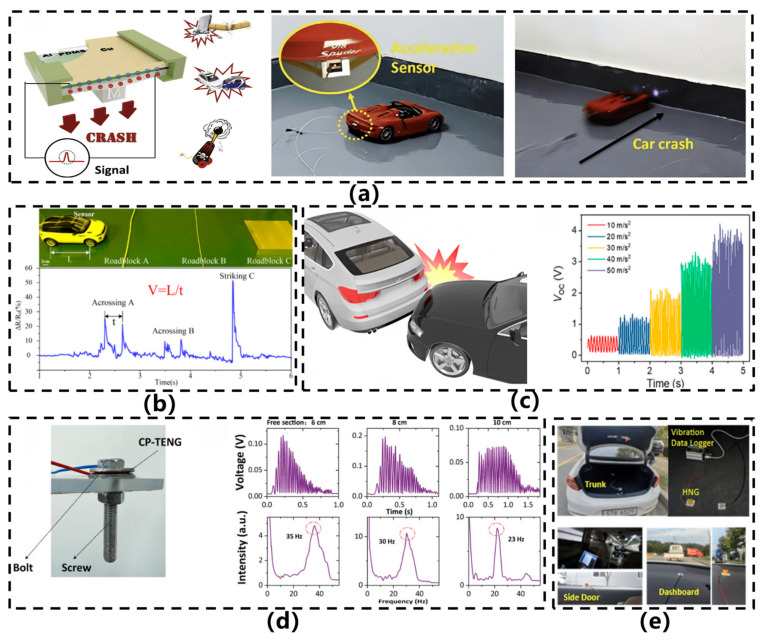
(**a**) Acceleration sensor employed for detecting vehicle collisions under high-g conditions [4]. (**b**) Vibration sensor is installed on the model car windshield, responding to vibrations from different road obstacles and calculating vehicle speed [25]. (**c**) Acceleration monitoring provides critical data to the airbag control unit for timely activation of protective devices [39]. (**d**) Flexible acceleration sensor is utilized for monitoring loosening of automotive bolts [41]. (**e**) Sensor identifying vehicle vibration characteristics and providing feedback on vehicle health status [55].

Keren et al. [4] proposed an acceleration sensor suitable for detecting car collisions under high-g conditions, specifically designed for extremely high-impact environments. It can accurately sense and measure extremely high accelerations (up to 18,000 g) without an external power supply. It can be applied in critical fields such as automotive collision safety monitoring and precise detonation control of missiles and ammunition. It can detect extremely high accelerations at the moment of collision in real time and reliably trigger safety devices such as airbags. In the military, it can accurately identify the acceleration characteristics of projectiles penetrating multiple obstacles, enabling intelligent detonation and target recognition. Compared with traditional piezoelectric sensors, this sensor greatly simplifies the signal processing process and has high linearity, as shown in Figure 13a. In 2021, Chen et al. [25] installed vibration sensors on the windshield of a model vehicle to monitor vibrations during collisions, as illustrated in Figure 13b. It can be demonstrated that these sensors respond to vibrations induced by various road obstacles, with resistance peaks accurately corresponding to the amplitude of vibrations generated. Furthermore, since the front and rear wheels of the vehicle produce two distinct vibration signals when passing over obstacles, the vehicle speed can be calculated by measuring the distance between the axles and recording the time interval between corresponding peaks. In 2021, the flexible acceleration sensor designed by Zhang et al. [39] was capable of simultaneously sensing acceleration changes in three-dimensional space. It could quickly identify the specific location and intensity of a collision when it occurred, thereby providing crucial data to the airbag control unit to ensure that protective devices were triggered promptly when necessary, effectively reducing the risk of occupant injury. Its miniaturized, lightweight, and highly integrated design also made it easy to embed in multiple key positions on the vehicle body, further enhancing the response accuracy and reliability of the vehicle’s overall safety system, as shown in Figure 13c.

In 2023, the flexible acceleration sensor proposed by Sun et al. can be used for monitoring the loosening of automotive bolts, as shown in Figure 13d [41]. This sensor can be directly integrated into the bolt washer and achieve self-powered operation by utilizing the vibration energy during vehicle travel. It can detect the vibration acceleration changes in real time to determine the bolt fastening status. When the bolt loosens, the sensor outputs characteristic high-voltage pulse signals, thereby providing early warnings for the safety status of key automotive components. This device has good temperature stability and mechanical durability, and can work reliably for a long time in complex vehicle-mounted environments, offering an intelligent solution for automotive bolt safety monitoring that does not require an external power supply, has a compact structure, and is highly responsive. In 2024, Bhatta et al. [55] introduced a flexible acceleration sensor capable of real-time monitoring of vehicle vibration states. Designed specifically for modern automotive Internet of Things (IoT) applications, this sensor efficiently converts low-amplitude, broadband vibrations generated during vehicle operation into electrical energy. The device can be installed in locations such as the trunk, dashboard, or inner door panels. Under varying road conditions—including smooth pavement, rugged terrain, and speed bumps—it not only provides a continuous power supply to low-power onboard IoT nodes, thereby extending battery life, but also accurately identifies vibration characteristics to deliver real-time road condition feedback and vehicle health monitoring for autonomous driving systems. Ultimately, the system forms a fully self-powered, wireless integrated platform for in-vehicle environmental monitoring and vibration sensing, as depicted in Figure 13e.

## 5. Conclusions

Flexible acceleration sensors have emerged as a transformative technology in healthcare, industrial monitoring, and consumer electronics, owing to their unique conformability and mechanical adaptability. This review systematically compares the two prevailing paradigms—hybridly flexible (rigid chip on flexible substrate) and intrinsically flexible (all-soft-material) sensors—and analyzes five core sensing mechanisms: capacitive, piezoresistive, triboelectric, piezoelectric, and electromagnetic. Each mechanism offers distinct trade-offs in sensitivity, frequency response, power consumption, and environmental robustness. For instance, capacitive sensors achieve high sensitivity (e.g., 0.197 pF/g with tunable range [30]), while piezoresistive devices exhibit ultra-wide bandwidth (0.1–20,000 Hz) and high gauge factors (up to 593.3 [25]). Triboelectric and piezoelectric types enable self-powered operation, with demonstrated sensitivities of 800 mV/g [39] and 4.558 V/g [5], respectively, and long-term durability exceeding 200,000 cycles [43]. Electromagnetic designs, though challenging to fully flexibilize, offer high linearity and passive wireless capability [8].

Despite these advances, practical deployment—especially in harsh environments like hydroelectric power plants [56]—remains constrained by critical challenges. High humidity, temperature fluctuations, and oil contamination accelerate material aging and interfacial delamination, while the low Young’s modulus (0.1–10 MPa) of soft materials limits inertial mass driving force, compromising sensitivity for low-amplitude vibrations (<0.2 g). Furthermore, most reported durability tests are limited to thousands of cycles [1], far below the billions required for real-world rotating machinery.

**Figure 14:**
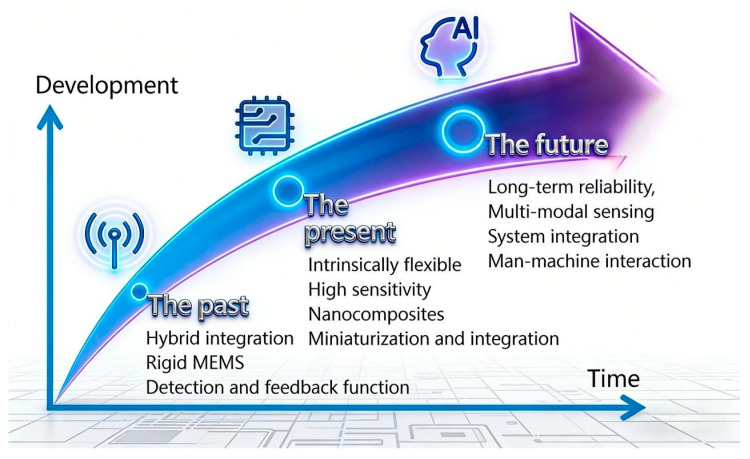
Technical roadmap of flexible acceleration sensor.

To guide future innovation, we envision a technology roadmap (Figure 14) that encapsulates the evolution of flexible acceleration sensors. In the past, research focused on hybrid integration (rigid MEMS chips on flexible boards) and proof-of-concept demonstrations using simple materials like paper and basic printing techniques. The present is characterized by intrinsically flexible materials (e.g., conductive polymers, liquid metals, nanocomposites), biomimetic microstructures (cracks, pores, pillars) [57] that boost sensitivity, and self-powering mechanisms (triboelectric/piezoelectric) that enable energy-autonomous operation. Looking to the future, the field will pivot toward environmental resilience (humidity/temperature resistance), long-term reliability (multi-million-cycle stability), multi-modal integration (combining acceleration with temperature, strain sensing), and system-level intelligence (on-chip processing, wireless connectivity, digital twins). These trends will drive the transition from laboratory prototypes to industrial-grade solutions, ultimately enabling predictive maintenance in critical infrastructure [1].

In summary, while flexible acceleration sensors have achieved remarkable performance milestones, bridging the gap between research and real-world application requires concerted efforts in material science, structural mechanics, and system engineering. By addressing the sensitivity–flexibility dilemma and enhancing environmental robustness, these sensors are poised to become cornerstone components in the Internet of Things and Industry 4.0, revolutionizing condition monitoring across energy, transportation, and healthcare sectors.

## Figures and Tables

**Table 1:** Summary and Comparison of Hybrid Flexible and Intrinsic Flexible Sensors.

Comparison	Dimension Hybridly Flexible Acceleration Sensor	Intrinsically Flexible Acceleration Sensor
Design Concept	Heterogeneous integration of “rigid chip + flexible carrier”	Unified design with “all-flexible materials”
Core Structure	Traditional rigid MEMS chip + flexible substrate/package (e.g., FPC, PI, PDMS)	Sensing element, electrodes, and substrate all composed of flexible materials
Material System	Silicon-based MEMS materials (rigid), metal interconnects, polymer substrates	Conductive materials (e.g., AgNWs, CNTs, LM), elastomers (PDMS, Ecoflex), functional composites
Key Fabrication	Chip thinning, flip-chip bonding, serpentine interconnects, flexible packaging	Micro/nano structuring (e.g., cracks, porosity), nanomaterial dispersion, transfer printing
Advantages	1. High and stable performance (inherits MEMS technology) 2. Relatively mature processes, easy integration 3. Comparatively low cost	1. Excellent conformability, adheres to complex curved surfaces 2. High deformation tolerance (bending, stretching, twisting) 3. Self-powering potential (piezoelectric/triboelectric types) 4. Lightweight, good biocompatibility
Limitations	1. Limited deformation threshold (semi-flexible) 2. Stress concentration at interfaces, prone to solder joint/interconnect fatigue fracture 3. Difficult to achieve high stretchability	1. Pronounced trade-off between sensitivity and flexibility 2. Low Young’s modulus of materials results in insufficient inertial driving force 3. Poor environmental stability (temperature/humidity), prone to aging 4. Low manufacturing consistency and standardization.
Typical Applications	Wearable health monitoring with moderate deformation requirements	Large deformation and complex curved surface scenarios, e.g., industrial equipment vibration monitoring, skin electronics

**Table 2:** Comparison of Different Acceleration Sensing Principles.

Sensing Type	Working Principle	Primary Advantages	Primary Disadvantages
Capacitive	Based on the change of parallel plate capacitance, the plate spacing or overlap area is changed by the displacement of the mass block.	High sensitivity, low power consumption, good temperature stability, low noise, and suitable for detecting weak and low-frequency signals.	Sensitive to environmental electromagnetic interference, may have signal drift, relatively complex structure.
Resistive	Based on the piezoresistive effect, the resistance of the material changes with stress/strain.	The principle is intuitive, the readout circuit is simple, the response is fast, the output signal is strong, and easy to integrate.	High temperature sensitivity, lag effect exists, high static measurement power consumption, and sensitivity is limited by the material.
Frictional electric	Based on contact electrification and the electrostatic induction effect, mechanical vibration directly converts into electrical signals.	Self-powered, simple structure, shock-resistant, suitable for extreme environments.	Output is easily affected by humidity, wear, and consistency of contact; sensitive to vertical direction, poor lateral coupling; signal stability is greatly affected by the environment.
Piezoelectric	Based on the piezoelectric effect, mechanical deformation generates charge.	High frequency response, high signal-to-noise ratio, no need for external power supply, suitable for dynamic vibration monitoring.	Low piezoelectric constant, prone to fatigue aging, insensitive to static acceleration, and material brittleness is relatively large.
Electromagnetic	Based on Faraday’s law of electromagnetic induction, changes in magnetic flux generate an induced electromotive force.	High sensitivity, good linearity, high reliability, and no need for an external power supply.	Large volume, high power consumption, difficult to be intrinsically flexible, complex integrated magnet or coil structure.

**Table 3:** Performance Comparison Table of Various Sensors.

Reference	Principle	Structure	Sensitivity	Linearity	Frequency Range	Cyclic Reliability
[3]	Piezoresistive	MWCNT/PDMS sandwich film	GF = 214.3 (at 0.4% bending strain)	-	25–100 Hz	Stable over 1000 bending (0.2% strain) and 1000 stretching (5% strain) cycles
[6]	Piezoresistive	Bio-inspired cilia-crack coupling	0.5 mV/g, GF up to 150	-	0–100 Hz	Stable over 1000 bending cycles (0.3% strain, 1 Hz), amplitude increase only 3%
[7]	Piezoresistive	MWCNT/CB/PDMS crack structure	GF = 2.46 (0–22% strain)	0.9812	up to 2000 Hz	Stable over 1000 cycles
[9]	Piezoresistive	Stainless steel sheet/PET double-sided tape/printing paper rigid-flexible composite sandwich structure	-	Good	up to 800 Hz	-
[10]	Piezoresistive	Four-beam kirigami structure, Ag/CNT strain sensor	z-axis: 0.064%/(m/s^2^) y-axis: 0.057%/(m/s^2^) x-axis: 0.00%/(m/s^2^)	Near-linear	1–6 Hz	-
[20]	Piezoresistive	Four-beam kirigami electrode structure, Ag/CNT strain sensor	<3 m/s^2^ (Detection threshold)	Near-linear	-	500 consecutive monitoring cycles
[24]	Piezoresistive	Paper-based cantilever beam	0.9 mV/mN	Good linearity	Not specified (Natural frequency ~ 15 Hz)	-
[25]	Piezoresistive	Channel crack suspended film, MWCNTs embedded in microchannels	GF up to 593.3 (3–5% strain)	Good linearity (acceleration response)	0.1–20,000 Hz	Stable over 10,000 cycles
[32]	Piezoresistive	Porous PDMS/Graphene foam	GF increased by 700% (Strain rate 10–1000 mm/min)	-	1–1000 Hz	Stable over 1000 cycles (1000 mm/min)
[33]	Piezoresistive	Bio-inspired radial bending micro-grooves (RACC arrangement)	GF > 1400 (0–0.46% strain), >18000 (0.46–0.65% strain)	-	70 Hz bandwidth	Stable over 7000 cycles
[34]	Piezoresistive	Parallel through cracks, Graphene/SIS	GF up to 657.36 (89–100% strain)	-	up to 103 Hz	Stable over 1000 cycles
[35]	Piezoresistive	Ag/PDMS/MWCNTs/CB structure, stainless steel ball mass	-	0.92	50–400 Hz	-
[5]	Piezoelectric	ZnO@PVDF composite nanofibers, sandwich structure	4.558 V/g	Good	20–50 Hz	Stable over 12,000 cycles
[16]	Piezoelectric	BaTiO_3_ nanowires embedded in P(VDF-TrFE) matrix	14 V, 4 µA	-	-	Stable over 10,000 cycles
[47]	Piezoelectric	Paper-based cantilever beam, U-shaped ZnO-NW coated paper	16.3 mV/g	-	84.75 Hz (Natural)	-
[49]	Piezoelectric	PZT/silicone rubber composite	8.59–9.81 mV/µε	Good	20–200 kHz	10^7^ cycles at 9200 µε; ~10^6^ cycles at 11000 µε
[11]	Capacitive	Ultra-thin perforated diaphragm array	270 mV/g	Linear	80–3400 Hz	-
[28]	Capacitive	Paper-based membrane structure, silver nano-ink sprayed electrodes	0.02 pF/g	Not specified	10–180 Hz (Test range)	-
[29]	Capacitive	Wheel-shaped hybrid film	20 mV/g	Linear	80–3000 Hz	-
[30]	Capacitive	3D seesaw structure, adjustable gap	0.197 pF/g (adjustable to 5.59 × 10^−3^ pF/g)	Linear	5–60 Hz (Low frequency)	Stable over 1000 cycles, stable over 100,000 s
[31]	Capacitive	Comb structure, roll-to-roll gravure printing	0.00133%/g (0–2.0 g)	Linear within 0–2.0 g	Not specified (Tested 0–3.6 g)	Max hysteresis error 31.49%
[17]	Triboelectric	PDMS pyramid microstructure	1.33 mV/(m/s^2^)	0.64%	80 Hz (Test frequency)	Stable performance after 15,000 g impact test
[22]	Triboelectric	Ecoflex/PEDOT:PSS, contact-separation mode	-	-	-	-
[23]	Triboelectric	Electrospun PVDF/BTO nanofibers	-	Linear 0–1.5 g	5–15 Hz	Stable over 5000 cycles
[26]	Triboelectric	Conductive sponge-silicon layer + FEP film	-	Fit: 0.954 (flat)/0.989 (curved)	10–100 Hz	Stable over 168,000 cycles
[27]	Triboelectric	Kirigami structure, PET/Cu/FEP	3.6 V/(m/s^2^) (1–5 m/s^2^), 17.5 V/(m/s^2^) (5.5–9 m/s^2^)	Good	2–49 Hz	Stable over 50,000 cycles
[37]	Triboelectric + Electret	Interdigitated electrodes, PTFE film, carbon fiber friction material	Voltage increased by 6 times (compared to uncharged)	Good	0.1–5 m/s^2^	Stable over 2400 s (~1000 cycles)
[38]	Triboelectric	Swim bladder membrane, copper nanoparticle electrodes	0.45 μA·s^2^/m (acceleration)	Acceleration R^2^ = 0.990	Test frequency 3 Hz	Stable over 850 cycles (1 Hz); Good output stability over 6 months
[39]	Triboelectric (Liquid Metal)	Mercury droplet, PTFE nanowire film	800 mV/g	Good vertical direction linearity	0–100 m/s^2^	No significant output attenuation after 100,000 cycles
[40]	Triboelectric	Sealed interconnected TENG array, biomimetic microstructure (Lotus leaf structure TPU + sandpaper rough silicone rubber)	0.37 V/kPa	Good	-	-
[41]	Triboelectric	CCTO/PDMS composite film, Kapton substrate	-	Good	9–60 m/s^2^	Stable over nearly 10,000 cycles
[42]	Triboelectric	Grid cavity structure, PU/PET/copper powder/PTFE powder	0.49584 mV/(m/s^2^)	-	8–6000 Hz	Stable over 35,000 cycles
[43]	Triboelectric (Liquid Metal)	Gallium-Indium-Tin liquid metal droplet	0.218 V·m^2^/s^2^	R^2^=0.995	1–5000 Hz	Stable over 216,000 cycles
[21]	MEMS Chip	Island-bridge structure, serpentine interconnects	-	-	2–6 Hz (Test range)	Stable under bending, stretching (10% strain), torsion
[18]	Electromagnetic (Magnetic Levitation)	Double-layer NdFeB/PDMS magnetic membrane, surface micropyramid array	0.82 mV/µm (120 Hz)	Sound pressure response R^2^ = 99.4%	1 Hz–20 kHz	Stable output after 4.5 million vibration cycles
[8]	Electromagnetic	Ring-shaped origami magnetic membrane (NdFeB/PDMS)	0.59 mV/µm (1.7 kHz)	-	1 Hz–10 kHz	Sensitivity decreased by 26.9% after 2000 bending cycles
[50]	Electromagnetic (LC Resonance)	Planar spiral inductor	0.27 mV/(m/s^2^)	Repeatability error < 0.037%	20–100 m/s^2^	-

## Data Availability

Not applicable.
